# Interconnected cell death pathways: central mechanisms and therapeutic targets in impaired follicular development of polycystic ovary syndrome

**DOI:** 10.1186/s13048-026-02054-5

**Published:** 2026-04-02

**Authors:** Shuang Ma, Chang Sun, Yasong Wang, Xuanning Zhang, Hongying Kuang

**Affiliations:** 1 Heilongjiang University of Chinese Medicine, Harbin, 150040 China; 2 Second Gynecology Department, The First Affiliated Hospital of Heilongjiang University of Chinese Medicine, No. 26 Heping Road, Xiangfang District, Harbin, 150040 China

**Keywords:** Polycystic ovary syndrome, Programmed cell death, Ovarian microenvironment imbalance, Pyroptosis, Inflammasome, Ferroptosis

## Abstract

Polycystic ovary syndrome (PCOS) is characterized by hyperandrogenism and insulin resistance, which synergistically disrupt the ovarian microenvironment. This pathological interplay induces chronic inflammation, oxidative stress, and metabolic dysregulation, subsequently triggering aberrant activation of multiple forms of programmed cell death (PCD) in granulosa cells, including apoptosis, pyroptosis, necroptosis, autophagy, and ferroptosis. Recent experimental studies, mainly based on animal and in vitro models, suggest that these death pathways may be interconnected through shared regulatory hubs, forming a dynamic interactive network referred to as the ‘death crosstalk’. Inhibition of apoptosis leads to defective follicular atresia and accumulation of immature follicles; pyroptosis and necroptosis amplify inflammatory cascades, promoting ovarian fibrosis and aggravating insulin resistance; ferroptosis induces lipid peroxidation–mediated granulosa cell injury, thereby reducing follicle-stimulating hormone (FSH) sensitivity; dysregulated autophagy exacerbates ferroptosis through ferritinophagy and degradation of GPX4. Targeting key nodes of the death crosstalk network—such as the NLRP3 inflammasome, RIPK1/RIPK3, and the ferroptosis–GPX4 axis—or employing epigenetic and gene-editing interventions, potentially holds promise for overcoming the limitations of single-target therapies. Such multi-target modulation may provide novel strategies to simultaneously improve ovulatory dysfunction and metabolic complications in PCOS.

## Introduction

Polycystic ovary syndrome (PCOS) is a common endocrine disorder involving the reproductive, metabolic, and dermatological systems, affecting 3–10% of women of reproductive age worldwide depending on diagnostic criteria [1]. The core pathological features of PCOS are hyperandrogenemia and insulin resistance, which synergistically induce chronic inflammation, oxidative stress, and metabolic dysregulation within the ovarian microenvironment. The ovarian microenvironment is a critical regulatory niche for follicular development and oocyte maturation, and its abnormal alterations in PCOS may represent a central factor leading to follicular developmental arrest and infertility. Specifically, PCOS-associated ovarian microenvironment abnormalities are characterized by a proinflammatory state (elevated IL-6 and TNF-α), oxidative stress (ROS accumulation), and metabolic dysregulation (abnormal androgen/insulin levels), collectively impairing granulosa cell function and disrupting follicular development [2]. Mechanistically, hyperandrogenemia exacerbates local inflammation through activation of the NF-κB pathway [3]; oxidative stress leads to mitochondrial dysfunction [4]; and insulin resistance further worsens the metabolic milieu [5]. Such an abnormal microenvironment can compromise follicular homeostasis by inducing granulosa cell death, ultimately resulting in dominant follicle selection failure and ovulatory dysfunction. From the perspective of folliculogenesis, the primary pathological hallmark of Polycystic Ovary Syndrome (PCOS) is not the premature depletion of the follicular reserve, but rather developmental aberrations during the transition from preantral to early- and mid-antral stages, as well as during dominant follicle selection [6]. This process is characteristically defined by the abnormal accumulation of small antral follicles, impaired dominant follicle selection, and subsequent anovulation. At these stages, granulosa cell proliferation, differentiation, and bidirectional communication with the oocyte are fundamental to follicular fate [7]. Distinct from classical follicular atresia, the sequestered small antral follicles in PCOS do not undergo rapid apoptotic clearance; instead, they persist in a state that is metabolically active yet developmentally restricted. This suggests that, beyond traditional apoptosis, alternative mechanisms of regulated cell death or sub-lethal injury may be involved [8]. The chronic inflammation, oxidative stress, and metabolic dysregulation associated with PCOS disrupt granulosa cell homeostasis during this critical window, trapping follicles in a “surviving but arrested” state rather than allowing them to proceed toward dominance or physiological atresia. This stage-specific developmental blockade provides a crucial pathological context for the aberrant activation and crosstalk of various regulated cell death pathways [9].

Recent studies have shown that the hyperandrogenic state in PCOS not only manifests as clinical features such as acne and hirsutism but also disrupts the ovarian microenvironment, interfering with dominant follicle selection [10]. Concurrently, metabolic disturbances such as insulin resistance in PCOS ovaries act synergistically with hyperandrogenemia to create a chronic inflammatory milieu, triggering aberrant activation of multiple granulosa cell death pathways, including apoptosis, pyroptosis, and autophagy [11, 12]. Importantly, these death pathways do not operate in isolation; they share regulatory molecules (ROS, AMPK/mTOR) and form a complex cross-regulatory network, termed “death crosstalk” [13], which cooperatively leads to extensive granulosa cell loss, accelerated follicular atresia, and ovulatory dysfunction [11]. However, current research on PCOS largely focuses on the regulatory mechanisms of single cell death pathways, and the dynamic interactions among multiple death modes and their synergistic pathogenic mechanisms remain underexplored.

This review summarizes current evidence—primarily derived from animal and in vitro models—regarding how “death crosstalk” exacerbates granulosa cell injury and follicular developmental defects. In contrast to previous reviews focusing on isolated cell death pathways, this article proposes an integrated “death crosstalk” model to conceptualize how apoptosis, pyroptosis, necroptosis, autophagy, and ferroptosis dynamically interact to collectively regulate granulosa cell survival, functional integrity, and follicular fate determination at specific developmental stages. This framework represents a novel conceptual advancement, providing a unified perspective that links reproductive, inflammatory, and metabolic abnormalities in PCOS. By highlighting the central regulatory nodes of this network, this review aims to establish a new theoretical foundation for multi-pathway synergistic therapies that transcend traditional single-target strategies.

## Literature search strategy

This review was conducted as a narrative synthesis aimed at conceptual integration rather than systematic evidence aggregation. A structured literature search was performed to ensure coverage of relevant studies.

Publications were retrieved from PubMed and Web of Science databases from inception to March 2025. Search terms included combinations of:“polycystic ovary syndrome” OR “PCOS” AND.

“apoptosis”, “pyroptosis”, “necroptosis”, “autophagy”, “ferroptosis”,as well as “ovary”, “granulosa cells”, and “folliculogenesis”.

Original research articles and reviews written in English and relevant to ovarian biology, reproductive endocrinology, or cell death signaling were considered. Priority was given to studies conducted in ovarian tissue, granulosa cells, or PCOS models. Mechanistic literature from other biological systems was included when necessary to contextualize pathway interactions.

Studies lacking mechanistic relevance, unrelated to reproductive biology, or duplicative in scope were excluded. Reference lists of selected papers were also screened to identify additional pertinent literature.

## Characteristics of key cell death and stress pathways in PCOS

**Fig. 1:**
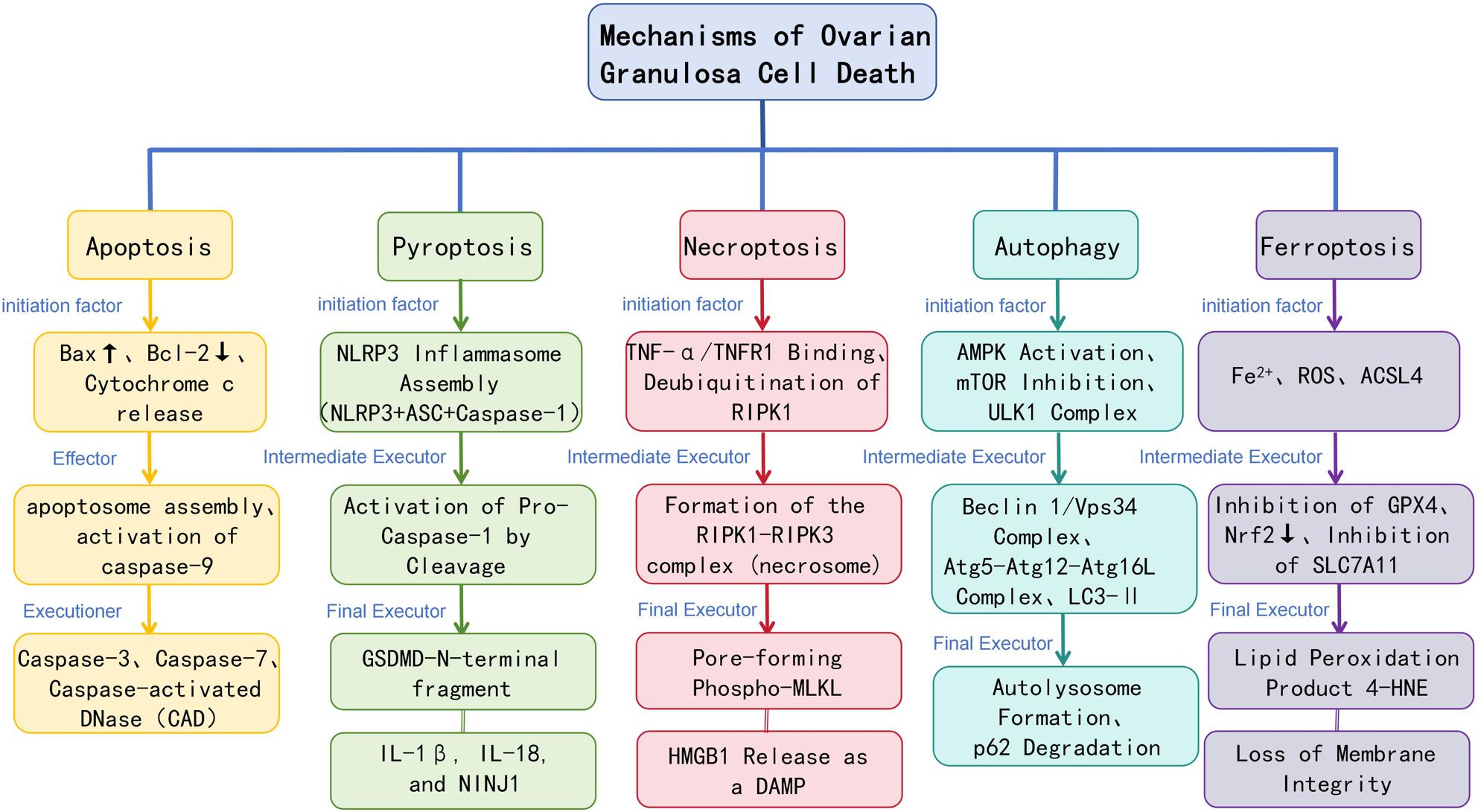
Conceptual overview of regulated cell death pathways in ovarian granulosa cells

In PCOS, ovarian dysfunction and follicular arrest are not driven by a single form of cellular injury, but rather by coordinated activation of multiple regulated cell death and stress-response pathways in granulosa cells. These pathways respond to metabolic overload, hyperandrogenism, oxidative stress, and chronic inflammation—hallmark features of the PCOS microenvironment—and collectively determine cell survival, follicle progression, or atresia. To provide a systematic overview of the molecular events discussed in this review, Fig. 1 summarizes the major regulated cell death programs implicated in granulosa cell dysfunction in PCOS. Rather than presenting a definitive or unified signaling model, this schematic serves as a conceptual framework that outlines the core features of each pathway and establishes a structural reference for the individual mechanisms discussed in the following subsections.

This figure provides a schematic overview of multiple regulated cell death programs that have been reported to be involved in granulosa cell dysfunction under PCOS-related conditions, including apoptosis, pyroptosis, necroptosis, autophagy-associated cell death, and ferroptosis. Each pathway is illustrated by its representative initiation signals, key molecular mediators, and terminal execution processes, as described in the literature. Apoptosis is characterized by caspase cascade activation following mitochondrial cytochrome c release. Pyroptosis involves NLRP3 inflammasome assembly, caspase-1 activation, gasdermin D–mediated pore formation, and pro-inflammatory cytokine release. Necroptosis is mediated by RIPK1–RIPK3 necrosome formation and subsequent MLKL-dependent membrane permeabilization. Autophagy-associated cell death is depicted by ULK1 activation and autophagosome–lysosome fusion, while ferroptosis is defined by iron-dependent lipid peroxidation associated with GPX4 inhibition and System Xc⁻ suppression. This schematic is intended as a conceptual reference to summarize pathway-specific features and to support the subsequent discussions, rather than to represent a definitive or exhaustive mechanistic model of granulosa cell death in PCOS.

### The role of apoptosis in PCOS: stage-specific regulation of follicular fate

Apoptosis is an essential programmed cell death mechanism for maintaining organismal homeostasis.The molecular mechanisms of apoptosis are induced by both intrinsic and extrinsic signals and are tightly regulated by caspase activity [14, 15]. While the core machinery involves the mitochondrial pathway (caspase-9) and the death receptor pathway (caspase-8) [16, 17], apoptotic regulation in PCOS is characterized by a significant spatiotemporal imbalance rather than a generalized enhancement. Under normal ovarian development, it occurs primarily within the granulosa cells (GCs) of antral and pre-ovulatory follicles. Under physiological conditions, apoptosis ensures the selection of a single dominant follicle and successful ovulation by selectively clearing non-dominant follicles.

However, evidence suggests that apoptotic regulation in PCOS is not merely a generalized enhancement but rather exhibits significant spatiotemporal imbalances. During the transition from preantral to early antral follicles—the critical window for the abnormal accumulation of follicles in PCOS—GCs often exhibit relatively suppressed apoptotic activity despite being exposed to metabolic and inflammatory stressors [18]. This resistance allows follicles to persist in a developmentally restricted state, contributing to the pathognomonic increase in small antral follicles. Mechanistically, hyperandrogenism and insulin resistance shift the balance between pro- and anti-apoptotic signaling [19]. Bas et al., through studies involving DHEA-induced PCOS rat models demonstrated that in PCOS granulosa cells, the expression of pro-apoptotic proteins (Bax) is significantly upregulated, while anti-apoptotic proteins (Bcl-2) are downregulated [20], leading to increased mitochondrial outer membrane permeabilization (MOMP) and promoting cytochrome c (Cytochrome C) release to activate the apoptosome. In this process, the caspase-9 and caspase-3 cascade ultimately triggers cell death [21]. Hyperandrogenemia in PCOS directly binds to androgen receptors (ARs), upregulating p53-dependent Bax expression while inhibiting Bcl-2 activity, thereby amplifying mitochondrial damage [22]. Moreover, the hyperandrogenic environment induces excessive reactive oxygen species (ROS) generation, further exacerbating mitochondrial DNA damage and apoptotic signaling [23]. Abnormal activation of the death receptor pathway also contributes to apoptosis regulation in PCOS. Cataldo et al., through immunohistochemical analysis and apoptotic cell detection in PCOS ovarian tissues [24], have shown that significantly elevated protein levels of Fas and FasL in ovarian tissues from PCOS patients; their interaction with Fas-associated death domain (FADD) initiates caspase-8-dependent extrinsic apoptosis [25]. Further studies indicate that proinflammatory factors within the local ovarian inflammatory microenvironment, such as TNF-α and IL-6, enhance death receptor signaling sensitivity via activation of NF-κB or JNK pathways [26]. In the early stages of follicular development, these signals may paradoxically promote granulosa cell survival; however, in later stages, they may predispose the follicles to aberrant apoptosis [27]. Taken together, apoptosis in PCOS is not uniformly enhanced but instead exhibits pronounced temporal and spatial dysregulation. Specifically, relative resistance to apoptosis in granulosa cells during the transition from preantral to early antral follicles may permit prolonged survival of metabolically active yet developmentally arrested follicles, contributing to the characteristic accumulation of small antral follicles [28]. In contrast, excessive or premature activation of apoptotic signaling at later stages may accelerate follicular atresia and impair dominant follicle selection, thereby disrupting ovulatory competence.

### The role of pyroptosis in PCOS: inflammatory amplification and follicular arrest

Pyroptosis, a proinflammatory death mode driven by the NLRP3–Gasdermin D (GSDMD) axis, plays a pivotal role in the chronic low-grade inflammation of PCOS [29]. In the ovarian microenvironment, the activation of the NLRP3 inflammasome complex triggers the cleavage of GSDMD, leading to N-terminal-mediated membrane pore formation and the subsequent secretion of IL-1β and IL-18 [30]. Furthermore, the terminal rupture of the plasma membrane, mediated by NINJ1, facilitates the massive release of damage-associated molecular patterns (DAMPs) such as HMGB1, thereby establishing a self-amplifying inflammatory loop that exacerbates ovarian dysfunction [31].

In PCOS, pyroptosis is thought to be closely associated with the chronic local ovarian inflammatory state and the resultant follicular arrest. This process is particularly prevalent in the granulosa cells of early- to mid-antral follicles, the critical developmental window characterized by the pathological accumulation of follicles in PCOS [32]. In PCOS, pyroptosis is activated through NLRP3 inflammasome activation driven by the synergistic effects of hyperandrogenemia and metabolic dysregulation [32]. Androgens promote the transcription of NLRP3 and pro-IL-1β via activation of the TLR4/NF-κB pathway, while oxidative stress (ROS) and endoplasmic reticulum stress (ERS) induced by insulin resistance accelerate inflammasome assembly [33]. Unlike classical apoptosis, which typically induces rapid cell clearance, pyroptosis in PCOS may manifest in a sublethal or focal manner. Rather than causing immediate follicular atresia, this process triggers a sustained release of pro-inflammatory cytokines that disrupts the delicate granulosa cell–oocyte bidirectional communication [34]. By maintaining a persistent inflammatory microenvironment, pyroptosis traps follicles in a characteristic “surviving but developmentally restricted” state, preventing their progression to dominance while avoiding definitive clearance [35]. Clinical studies have shown that expression of the GSDMD N-terminal fragment is significantly elevated in granulosa cells from PCOS patients, and serum IL-1β levels positively correlate with follicular atresia rates. This suggests a fundamental link between pyroptotic signaling and the aberrant determination of follicular fate. Furthermore, free fatty acids (FFAs) in the metabolic microenvironment and lipopolysaccharide (LPS) translocation caused by gut dysbiosis can enhance NLRP3 inflammasome activity through the CD36/TLR4 signaling pathway, forming a vicious “metabolic abnormality–inflammation–pyroptosis” cycle [36]. It should be noted that most direct evidence regarding NLRP3 inflammasome activation in the ovary is currently derived from DHEA-induced PCOS animal models and in vitro experiments; its stage-specific role in human ovarian tissue remains to be further validated.Collectively, current evidence suggests that pyroptosis in PCOS primarily functions as an inflammatory amplifier within the ovarian microenvironment rather than as a direct executor of follicular atresia.

### The role of necroptosis in PCOS: inflammation-driven ovarian structural remodeling

Necroptosis, a regulated form of necrotic cell death, is increasingly recognized as a potent driver of ovarian chronic inflammation and structural remodeling. Its core pathway is activated by TNF family receptors, Toll-like receptors (TLRs), or pathogen-sensing receptors [37]. Unlike survival-oriented signaling, the inhibition of caspase-8 shifts the TNFR1-mediated complex toward the formation of the “necrosome,” where RIPK1 recruits RIPK3 to phosphorylate MLKL [38]. The subsequent translocation of phosphorylated MLKL to the plasma membrane induces pore formation and the explosive release of DAMPs. In the context of PCOS, this necroptotic cascade not only triggers localized immune responses but also promotes fibroblast activation and abnormal extracellular matrix (ECM) deposition, which are fundamental to ovarian fibrosis and follicular arrest [39, 40].

In contrast to apoptosis, which is primarily and directly involved in follicle selection and atresia, necroptosis in PCOS is more likely to indirectly influence the follicular microenvironment. This effect is mediated through its potent pro-inflammatory and tissue-remodeling properties, which exert a particular impact on the ovarian interstitium and the peri-follicular stroma. In the pathological progression of the PCOS ovary, persistently elevated proinflammatory factors in the ovarian microenvironment, such as TNF-α and IL-6, can activate the canonical TNFR1 signaling pathway, causing dissociation of the RIPK1–TRADD/FADD complex. Under conditions of inhibitory ubiquitination deficiency, for example, when caspase-8 activity is blocked, RIPK1 undergoes autophosphorylation and recruits downstream RIPK3 [41]. The resulting amyloid-like complex (necrosome) further phosphorylates MLKL, triggering its oligomerization and translocation to the plasma membrane to form pores, ultimately leading to cellular swelling and leakage of contents [42].In PCOS ovarian tissues, the accumulation of reactive oxygen species (ROS) associated with insulin resistance may further sensitize the necroptotic pathway via oxidative modification of RIPK1. At the functional level, necroptosis may drive the structural abnormalities of the PCOS ovary through a dual “inflammation–fibrosis” mechanism: on one hand, DAMPs bind to TLRs or the NLRP3 inflammasome, inducing macrophage polarization toward a proinflammatory (M1) phenotype and promoting secretion of profibrotic factors such as TGF-β1 and IL-1β [43, 44]; on the other hand, MLKL activation directly upregulates collagen synthesis-related genes and profibrotic mediators in ovarian stromal cells and induces stromal cells to transdifferentiate into myofibroblasts via activation of the MAPK/ERK and NF-κB pathways [45]. This necroptosis-driven “inflammation-fibrosis” vicious cycle may be closely linked to ovarian enlargement, cortical thickening, and ovulatory dysfunction in patients with PCOS, thereby spatially restricting follicular expansion and dominant follicle selection [46].It must be emphasized that current direct evidence regarding the role of necroptosis in the PCOS ovary is primarily derived from cell culture systems and rodent models. Its precise anatomical localization, cell-type specificity, and stage-dependent characteristics in human ovarian tissue remain to be systematically elucidated through further research. Overall, necroptosis in PCOS is more likely to influence follicular fate indirectly by reshaping the ovarian microenvironment rather than determining the survival of individual follicles.

### The role of autophagy in PCOS: granulosa cell homeostasis and follicular arrest

Autophagy is a highly conserved lysosomal-dependent intracellular degradation pathway in eukaryotic cells. By clearing damaged organelles, misfolded proteins, and metabolic waste, it plays a critical role in maintaining cellular homeostasis and facilitating adaptation to environmental stress [47]. During normal folliculogenesis, autophagy is considered essential for the metabolic adaptability of granulosa cells, mitochondrial quality control, and the functional coupling between the oocyte and granulosa cells. This is particularly evident during the transition from preantral to antral follicle development, where its moderate activation supports follicular growth rather than inducing cell death [48]. In ovarian dysfunction associated with PCOS, autophagy exhibits dynamic bidirectional regulation. In the early stages of disease or during the initial phase of stress, autophagy in granulosa cells may be transiently activated as a compensatory protective mechanism to counter the primary insults of hyperandrogenism, metabolic stress, and oxidative stress [49]. However, as hyperandrogenism and insulin resistance persist, the chronic inhibition of the PI3K/Akt/mTOR signaling pathway leads to decreased Beclin-1 expression and impaired autophagic flux, ultimately compromising the cytoprotective role of autophagy in granulosa cells [50]. Functionally, autophagic dysfunction does not necessarily trigger classical cell death; rather, it likely maintains follicles in a “surviving but functionally impaired” state by disrupting mitochondrial quality control and cellular metabolic homeostasis [51]. Existing studies suggest that defects in PINK1/Parkin-mediated mitophagy in PCOS lead to the excessive accumulation of reactive oxygen species (ROS) [52]. This further amplifies the local inflammatory response via the ROS–NLRP3 inflammasome axis, thereby interfering with granulosa cell–oocyte communication and promoting follicular arrest rather than rapid atresia [50]. In the context of PCOS, dysregulated autophagy in granulosa cells appears to support cell survival under metabolic and oxidative stress while simultaneously impairing proper follicular progression. This dual effect may favor the persistence of developmentally arrested follicles rather than their timely elimination via physiological atresia. It should be noted that most current evidence regarding the stage-specific role of autophagy in the PCOS ovary is primarily derived from animal models and in vitro studies of cultured granulosa cells. Whether autophagy exerts a protective or a pro-pathological effect at different stages of follicular development, and its precise regulatory mechanisms in the human ovary, remain to be systematically elucidated.

### The role of ferroptosis in PCOS: lipid peroxidation-driven irreversible follicular damage

Ferroptosis is an iron-dependent, lipid peroxidation-driven form of programmed cell death, characterized by collapse of the cellular antioxidant defense system and excessive accumulation of lipid reactive oxygen species (ROS) [53]. Mechanistically, inhibition of System Xc⁻depletes intracellular glutathione (GSH) [54], leading to the inactivation of glutathione peroxidase 4 (GPX4) and subsequent accumulation of toxic lipid hydroperoxides [55]. Concurrently, free ferrous ions (Fe²⁺) catalyze lipid peroxidation via the Fenton reaction [56]. This process ultimately triggers plasma membrane rupture and mitochondrial damage, promoting the release of damage-associated molecular patterns (DAMPs) [57].

In the physiological context of folliculogenesis, ferroptosis is thought to occur more frequently during the stages of antral follicle development and dominant follicle selection—phases that are metabolically active and highly sensitive to oxidative stress. Granulosa cells (GCs) at this stage rely on precise lipid metabolism and antioxidant homeostasis to maintain their responsiveness to follicle-stimulating hormone (FSH), making them particularly vulnerable to lipid peroxidative damage [58]. In the pathological condition of PCOS, the ovarian microenvironment—characterized by the accumulation of free iron, suppression of GPX4 expression, and persistent oxidative stress—creates favorable conditions for the occurrence of ferroptosis [59]. Functionally, rather than causing the long-term arrest of small follicles, ferroptosis is"more likely to drive f"llicular atresia during the critical window of transition from antral to dominant follicles by inflicting irreversible damage on GCs [60]. Lipid peroxidation products characteristic of ferroptosis (4-HNE) can covalently modify follicle-stimulating hormone receptor (FSHR), reducing granulosa cell sensitivity to FSH, while impairing CYP17A1 activity in theca cells, exacerbating the hyperandrogenism–insulin resistance vicio"s cycle and triggering"release of inflammatory DAMPs such as HMGB1. This forms an “oxidative–inflammatory” vicious cycle [61]. In terms of pathological consequences, ferroptosis may also participate in the structural abnormalities of the PCOS ovary through the “oxidative stress–inflammation–tissue remodeling” axis [62]. Animal studies have demonstrated that the ferroptosis inhibitor ferrostatin-1 significantly alleviates ovarian fibrosis in DHEA-induced PCOS rats. This is evidenced by the downregulated expression of Col1a1 and α-SMA, suggesting that ferroptosis exerts a pro-pathological role in ovarian interstitial remodeling [63]. Taken together, ferroptosis in PCOS is more likely to be engaged during metabolically active antral stages and the critical window of dominant follicle selection, where granulosa cells are highly dependent on intact lipid redox homeostasis [62]. Rather than sustaining long-term follicular arrest, ferroptosis-associated lipid peroxidation may drive irreversible granulosa cell damage, promote follicular atresia, and exacerbate ovarian structural remodeling [59]. However, it must be emphasized that most current evidence regarding the role of ferroptosis in PCOS is derived from rodent models; whether ferroptosis is activated at specific follicular stages in human PCOS ovarian tissue, as well as its spatial distribution and clinical relevance, remains to be further validated.

## Crosstalk mechanisms among cell death pathways

However, it must be emphasized that most current evidence regarding the role of ferroptosis in PCOS is derived from rodent models; whether ferroptosis is activated at specific follicular stages in human PCOS ovarian tissue, as well as its spatial distribution and clinical relevance, remains to be further validated.As summarized in Table 1, the evidence for different PCD modes in PCOS is hierarchical. While apoptosis and pyroptosis are supported by clinical data, the roles of necroptosis and their mutual crosstalk remain largely inferential, primarily extrapolated from non-ovarian disease models.

**Table 1:** Evidence hierarchy and mechanistic insights into Programmed Cell Death (PCD) in PCOS

PCD Mode/Crosstalk Hub	Key Biomarkers	Direct Human Evidence (Clinical)	PCOS-specific Experimental Evidence (In Vivo/In Vitro)	Hypothetical & Cross-disease Inference
Apoptosis	Caspase-3/8/9, Bax, Bcl-2, Fas/FasL	Increased Bax/Bcl-2 ratio in GCs; elevated FasL levels in follicular fluid (FF).	DHEA or Letrozole-induced rodent models show follicular atresia linked to Caspase cascade activation.	Well-established mitochondrial and death receptor pathways in follicular arrest.
Pyroptosis	NLRP3, GSDMD-NT, IL-1β, IL-18	Upregulated NLRP3 and GSDMD-NT in GCs; elevated pro-inflammatory cytokines (IL-1β) in FF.	Hyperandrogenism and hyperinsulinemia trigger ROS-mediated NLRP3 activation in cultured GCs.	ROS-TXNIP-NLRP3 axis as a universal amplifier of metabolic inflammation (extrapolated from metabolic diseases).
Ferroptosis	GPX4, SLC7A11, ACSL4, Lipid ROS	Decreased GPX4 expression and accumulation of lipid peroxidation products in GCs of PCOS patients.	Ferrostatin-1 treatment attenuates ovarian fibrosis and restores follicular development in DHEA-treated rats.	ACSL4-mediated lipid peroxidation is hypothesized to impair FSH receptor sensitivity (mechanistic inference).
Autophagy	LC3B-II/I, Beclin-1, p62, Autophagosomes	Increased LC3B-II/I ratio and presence of autophagic vacuoles in clinical GC samples.	Autophagy acts as a survival mechanism under stress (hypoxia/IR) but promotes GC loss when overactivated.	Autophagy-dependent ferroptosis (ferritinophagy) as a potential mechanism for iron overload in follicles.
Necroptosis	RIPK1, RIPK3, p-MLKL	Limited/Lacking.Minimal studies reporting RIPK1 fluctuations in human PCOS follicles.	Increased p-MLKL expression in DHT-induced PCOS rats; RIPK1 inhibitors (e.g., Nec-1) partially rescue phenotype.	Transition from apoptosis to necroptosis under Caspase-8 inhibition (inferred from cancer and inflammatory models).
Death Crosstalk	p53, ROS, HMGB1	Very Limited. Fragmentary observations of concurrent pathway activation in patient GCs.	Compensation/switch between death modes (e.g., inhibition of apoptosis inducing pyroptosis) in experimental models.	ROS and p53 proposed as central hubs coordinating multiple PCDs (inferred from ischemia-reperfusion injury models).

### Receptor-mediated death crosstalk: apoptosis, pyroptosis, necroptosis

#### Central hub role of the RIPK1–RIPK3–MLKL axis

The RIPK1–RIPK3–MLKL signaling axis, as the core execution pathway of necroptosis, participates in the crosstalk among programmed cell death pathways, including apoptosis and pyroptosis, through multi-layered regulatory interactions [64]. Its central function lies in RIPK3-mediated phosphorylation of MLKL, which triggers MLKL oligomerization and membrane translocation, forming transmembrane pores that disrupt plasma membrane integrity and ultimately induce osmotic cell death (necroptosis) [65]. This pathway has a bidirectional regulatory relationship with apoptosis: when caspase-8 activity is inhibited (by viral-encoded inhibitors) or absent, the stability of the RIPK1–RIPK3 complex is enhanced, driving MLKL-dependent necroptosis [66]; conversely, caspase-8 can cleave RIPK1/RIPK3 to block this pathway, shifting the death mode toward apoptosis [67]. Furthermore, K⁺ efflux mediated by MLKL pores can activate the NLRP3 inflammasome, promoting caspase-1-dependent GSDMD cleavage and establishing indirect necroptosis-to-pyroptosis crosstalk [68]. Notably, RIPK1 functions as a molecular switch, dynamically regulating cell fate through complex transitions: the pro-survival Complex I (TNFR1–TRADD–TRAF2–RIPK1) activates the NF-κB pathway [69], whereas the pro-death Complex II (RIPK1–RIPK3–caspase-8) determines the initiation of apoptosis or necroptosis [70].

In the complex pathological network of PCOS, the RIPK1–RIPK3–MLKL axis is hypothesized to act as a central hub, orchestrating different modes of programmed cell death to drive its key pathologies, including abnormal follicular development, a pro-inflammatory microenvironment, insulin resistance, and hyperandrogenism [19, 71]. It should be noted that current evidence regarding the role of this signaling axis in PCOS is primarily derived from in vitro cell models and rodent studies; its functional role as a unified ‘central hub’ in the human PCOS ovary still lacks direct functional validation. As a critical molecular switch, RIPK1 under specific stimuli tends to suppress its pro-apoptotic kinase activity and promotes formation of the pro-survival Complex IIa with FADD/caspase-8, or directly inhibits complete caspase-8 activation, thereby impeding granulosa cell apoptosis [72]. It should be emphasized that this inference is primarily based on the established physiological principle that the ‘inhibition of granulosa cell apoptosis can reduce follicular atresia.’ Currently, direct evidence is lacking to demonstrate that RIPK1 predominantly dictates follicular fate in this manner within PCOS ovarian granulosa cells; its role is more likely a single link within a multi-factorial regulatory network. Normally, granulosa cell apoptosis is a key step in follicular atresia; its inhibition directly reduces follicular atresia, allowing developmentally arrested follicles to persist and accumulate, forming the polycystic ovarian morphology characteristic of PCOS. In this context, RIPK1’s “death-switching” function favors survival and represents an upstream molecular event for abnormal follicle accumulation. Simultaneously, RIPK1/RIPK3 activation phosphorylates and activates MLKL. Activated MLKL can oligomerize and disrupt the plasma membrane, directly inducing pyroptosis; it can also significantly activate the NLRP3 inflammasome [73]. Activated MLKL can oligomerize and disrupt the plasma membrane, inducing necrotic cell lysis and significantly promoting NLRP3 inflammasome activation through mechanisms such as K+ efflux, thereby indirectly amplifying the pyroptotic response [68]. Inflammasome assembly activates caspase-1, which cleaves GSDMD to generate the pore-forming N-terminal fragment (GSDMD-NT), synergistically amplifying pyroptotic effects [74]. In ovarian macrophages, excessive activation of this axis leads to macrophage pyroptosis. Ruptured pyroptotic cells release abundant proinflammatory factors, particularly IL-1β [75]. IL-1β establishes a local chronic low-grade inflammatory microenvironment in the ovary, which interferes with insulin signaling and serves as an important driver of ovarian insulin resistance in PCOS, thereby affecting follicular development and hormone synthesis [76]. Furthermore, although relevant evidence is primarily derived from non-ovarian cell models (such as fallopian tube epithelial cells), RIPK3 can directly induce GSDMD-mediated pyroptosis under specific conditions, independent of classical caspase cleavage. This mechanism suggests a more direct connection between necroptosis and pyroptosis [77]. This highlights RIPK3’s direct executor role in the pyroptotic pathway, expanding the functional versatility of this axis. Pyroptosis of fallopian tube epithelial cells can propagate inflammation beyond the ovary, causing cytokine leakage, immune cell infiltration, and tissue damage, ultimately disrupting the pelvic microenvironment. Such pelvic disturbance may impair oocyte transport, maturation, and final quality, correlating with reduced fertility in PCOS patients. Although these findings require further validation within ovarian systems, they provide potential clues for understanding the dissemination of inflammatory signals within the reproductive tract [78, 79]. Notably, the aberrant signaling of the RIPK1–RIPK3 axis is also an important upstream regulator of the NF-κB pathway. Its persistent activation may promote the NF-κB-dependent expression of androgenic enzymes in theca cells, thereby establishing a potential link with hyperandrogenism in PCOS [80, 81], the RIPK1–RIPK3–MLKL axis likely participates in the formation of abnormal follicular development and ovarian microenvironmental remodeling in PCOS by integrating cell death, inflammation, and metabolic signals.

#### Death decision switch function of caspase-8

Among the signaling mediators coordinating death-mode switching, caspase-8 represents a crucial control node in the death crosstalk network. Caspase-8 functions as a molecular decision hub in programmed cell death, dynamically balancing the activation thresholds of apoptosis, necroptosis, and pyroptosis, thereby driving crosstalk among cell death modes.

In the apoptotic pathway, activation of death receptors (Fas/TNFR1) recruits FADD to form the DISC (Death-inducing signaling complex), inducing autocatalytic activation of caspase-8 and triggering downstream caspase-3/7-dependent apoptosis, enabling orderly cell clearance [82].Concurrently, caspase-8 cleaves RIPK1 (at D324) and RIPK3 (at D328), inhibiting their kinase activities and preventing RHIM domain-mediated necrosome (RIPK1–RIPK3–MLKL) assembly, thereby serving as a molecular brake on necroptosis [83]. In pyroptotic regulation, caspase-8 exhibits a bidirectional role: under homeostatic conditions, it cleaves the pyroptotic effector GSDMD to generate an inactive C-terminal fragment (GSDMD-C), suppressing plasma membrane pore formation [84]; under inflammatory stimulation, caspase-8 activates the NLRP3 inflammasome via the ASC adaptor protein, promoting caspase-1-dependent cleavage of GSDMD-N and amplifying pyroptotic effects [85]. The essence of this functional state lies in its death decision: sufficient caspase-8 activity preferentially initiates apoptosis while inhibiting necroptosis/pyroptosis.

In the pathological environment of PCOS, evidence from Zanjirband et al., based on bioinformatic screening of multiple GEO datasets and subsequent validation in human granulosa cells, suggests that caspase-8 activity may be suppressed by the synergistic effects of hyperandrogenism and insulin resistance in granulosa cells during the early-to-mid antral follicle stages [25]. This inhibition may be achieved through the AR–p53 signaling axis and JNK-mediated phosphorylation at the S380 site, thereby compromising the catalytic activity of caspase-8 [25, 86].

In this context, the protective cleavage of RIPK1/RIPK3 is obstructed, which may promote the parallel or cross-activation of necroptosis and NLRP3-dependent pyroptosis. Consequently, the inhibition of apoptosis resulting from caspase-8 suppression triggers uncontrolled necroptosis, manifested by the MLKL phosphorylation-mediated release of damage-associated molecular patterns (DAMPs) [87]. These DAMPs are thought to further amplify inflammasome activation and IL-1β-mediated local inflammatory responses, forming a potential positive feedback loop that impairs the process of follicular development and exacerbates metabolic imbalance [19, 88]. In granulosa cells from PCOS patients, caspase-8 gene expression is significantly reduced [89], resulting in dysfunction of this death decision hub and imbalance in crosstalk among multiple death pathways: apoptotic blockage impairs DISC assembly, reducing physiological clearance of granulosa cells and leading to follicular atresia failure and persistent accumulation of immature follicles [12]; uncontrolled necroptosis arises from failed RIPK1/RIPK3 cleavage, enhancing MLKL phosphorylation, inducing granulosa cell membrane rupture, and releasing DAMPs (HMGB1), which activate macrophage TLR4 signaling to secrete TGF-β1 and drive ovarian collagen deposition [90]. The pyroptotic cascade is amplified via weakened GSDMD inhibition and aberrant NLRP3 inflammasome activation, promoting massive IL-1β/IL-18 release, which impairs local insulin signaling and suppresses GDF9 secretion from cumulus cells, further compromising oocyte maturation [91].

Taken together, current evidence supports a model in which dysregulated caspase-8 activity may serve as a critical junction connecting metabolic stress and abnormal follicular development in PCOS. Its functional restriction or shift may tilt the cell death pattern away from apoptosis and toward necroptosis and pyroptosis, thereby contributing to the development of follicular arrest, ovarian remodeling, and metabolic disturbances. It should be noted that the aforementioned shift in death modes and the ensuing inflammatory consequences triggered by caspase-8 inhibition are currently based on inferences from inflammatory diseases and PCOS animal models; whether this forms a stable positive feedback loop in the human PCOS ovary remains to be validated by further research.

#### Execution and crosstalk regulation of gasdermin protein family (GSDM)

The Gasdermin protein family (GSDMD/GSDME) serves as a “pore-forming execution hub” in programmed cell death, mediating core pyroptotic effects, bridging apoptosis–pyroptosis conversion, and amplifying necroptotic inflammatory signaling, thus driving dynamic interplay among multiple death pathways. In the pyroptotic pathway, caspase-1 activated by the NLRP3 inflammasome specifically cleaves GSDMD, releasing its N-terminal fragment (GSDMD-N), which oligomerizes to form plasma membrane pores, inducing cellular swelling and the release of proinflammatory cytokines such as IL-1β and IL-18 [92]. Meanwhile, in apoptosis-related contexts, caspase-3 or caspase-8 can cleave GSDME, shifting the predominantly apoptotic death process toward a GSDME-dependent pyroptosis-like phenotype. This mode switching is particularly pronounced in pathological environments characterized by restricted caspase-3 activity or an enhanced inflammatory background [93]. Critically, the GSDM family integrates the death network through multilayered crosstalk: necroptotic effector MLKL-mediated K⁺ efflux activates the NLRP3 inflammasome, promoting caspase-1-dependent GSDMD cleavage; ferroptosis-specific lipid peroxidation product 4-HNE covalently modifies GSDMD-Cys191, significantly enhancing pore activity, forming a “ferroptosis→pyroptosis” positive feedback loop, collectively constituting the terminal execution hub of death crosstalk [94].

In PCOS-related studies, Gasdermin-mediated pyroptotic signaling has been observed to primarily involve ovarian granulosa cells and cumulus cells, potentially influencing follicular fate during the early-to-mid antral stages [12]. Experimental models demonstrate that GSDME-dependent pyroptosis-like death in granulosa cells is associated with irreversible cell loss, with electron microscopy revealing morphological features where apoptotic bodies coexist with pyroptotic membrane pores [95]. Beyond the inhibitory effects of pyroptotic IL-1β on GDF9, studies by Miyoshi et al. using co-culture bioassays indicate that the KL–c-kit signaling axis is a critical regulator of the oocyte-granulosa cell communication niche. Specifically, they found that endogenous oocyte factors, particularly GDF-9 and FGF-8, are significantly altered following KL treatment, leading to the suppression of FSH-induced aromatase mRNA expression and estradiol synthesis. This provides further evidence that any disruption to GDF9 signaling—whether via inflammatory pyroptosis or altered KL-c-kit interaction—compromises the follicular microenvironment [96]. At the level of ovarian structural remodeling, the GSDME-N fragment has been found to activate the TGF-β1/Smad3 signaling pathway in ovarian stromal fibroblasts, inducing the upregulation of COL1A1 and COL3A1 expression. In PCOS mouse models, GSDME deficiency is associated with reduced ovarian collagen deposition and a significant alleviation of fibrosis [97–99]. Furthermore, GSDMD-mediated pyroptosis may also influence systemic immuno-metabolic pathways, such as hepatic inflammation and the regulation of metabolic homeostasis, through the release of HMGB1 [100]. Integrating the current evidence, Gasdermin-mediated pore formation events may contribute to the accelerated follicular atresia and associated metabolic abnormalities in PCOS through a cascade axis of ‘local follicular pyroptosis—ovarian stromal remodeling—systemic metabolic stress‘ [101]. However, it should be noted that the spatiotemporal-specific regulation and cell-type-dependent roles of Gasdermin signaling in PCOS are still primarily inferred from experimental models and require more direct validation in human ovarian tissues.

### Apoptosis and ferroptosis

#### The central hub of endoplasmic reticulum (ER) stress

Endoplasmic reticulum (ER) stress acts as a critical hub for apoptosis–ferroptosis crosstalk, coordinating both programmed cell death processes via the unfolded protein response (UPR) signaling pathway [102]. Ferroptosis inducers (erastin, RSL3) disrupt ER homeostasis, activating the PERK–eIF2α–ATF4–CHOP signaling axis: ER stress first triggers PERK kinase activation, leading to eIF2α phosphorylation, which inhibits global protein translation while selectively promoting transcription factor ATF4 expression. ATF4 subsequently upregulates the pro-apoptotic factor CHOP (C/EBP homologous protein) [103]. CHOP activates PUMA (p53 upregulated modulator of apoptosis) in a p53-independent manner, driving Bax/Bak-dependent mitochondrial outer membrane permeabilization (MOMP) and initiating the caspase cascade to induce classical apoptosis [104, 105]. Additionally, ER stress suppresses GPX4 activity or expression via the ATF4–CHOP axis, significantly impairing antioxidant capacity, promoting lipid peroxidation accumulation, and amplifying ferroptotic signaling [102, 106]. Under pathological stress conditions, this CHOP–PUMA axis may facilitate the synergistic enhancement of apoptosis and ferroptosis signals, rather than occurring independently. This provides a potential molecular foundation for multi-pathway intervention strategies.

In PCOS, growing evidence suggests that ER stress mediated by the CHOP–PUMA axis is involved in the pathogenesis and progression of ovarian dysfunction [107]. Characteristic PCOS pathological changes—including hyperandrogenism, insulin resistance, and chronic oxidative stress—synergistically disrupt granulosa cell ER homeostasis, inducing calcium imbalance and protein misfolding, persistently activating the PERK–ATF4–CHOP pathway. This pathway upregulates PUMA expression, driving granulosa cell apoptosis [108, 109]. Evidence from Liu et al., based on integrated in vitro cell assays and in vivo oxidative stress models, indicates that PUMA expression positively correlates with follicular atresia. Their study demonstrated that oxidative stress induces PUMA upregulation via JNK-dependent activation of FoxO1, providing strong molecular evidence that this signaling cascade drives granulosa cell apoptosis in the pathological ovarian microenvironment [110]. Recently published studies have demonstrated an association between the FoxO pathway and PCOS. Reports indicate that the expression of FoxO1—a member of the FoxO subfamily expressed in nearly all human tissues—is significantly elevated in the cumulus cells of women with PCOS [25]. Separately, studies in animal models suggest that hyperandrogenic microenvironments can downregulate hepcidin, increasing the labile iron pool, which synergizes with ER stress–mediated GPX4 inhibition to promote lipid peroxidation [111]. This process may trigger granulosa cell ferroptosis and accelerate ovarian reserve decline; however, direct evidence for this hepcidin–ferroptosis pathway in human PCOS ovarian tissues is currently lacking and requires further investigation.

#### Bidirectional regulatory mechanism of p53

p53 functions as a pivotal molecular switch, dynamically coordinating apoptosis and ferroptosis by differentially regulating downstream targets. At the transcriptional level, p53 activates pro-apoptotic genes (PUMA, BAX, and NOXA), inducing mitochondrial outer membrane permeabilization (MOMP) and triggering the caspase cascade to initiate classical apoptosis. Concurrently, p53 directly suppresses the expression of SLC7A11, a key component of the cystine/glutamate antiporter (System Xc⁻), reducing cystine uptake and intracellular glutathione (GSH) synthesis, thereby weakening antioxidant defenses and promoting ferroptosis [112, 113]. This bidirectional regulation is context-dependent: under genotoxic stress such as DNA damage, p53 phosphorylation via ATM/ATR kinases (Ser15) preferentially activates apoptotic pathways [114]; under persistent oxidative stress, p53 can enhance ferroptosis sensitivity through transcription-independent mechanisms [115], allowing dynamic switching between death modalities.

In PCOS, dysregulation of the p53 pathway within the ovarian microenvironment is thought to play a potential role in aberrant granulosa cell death. Hyperandrogenism-induced chronic oxidative stress markedly increases p53 phosphorylation in granulosa cells. Through an integrated approach of bioinformatic re-analysis and clinical validation, Zanjirband et al. [25] demonstrated the critical role of the p53 signaling pathway in PCOS. All comparisons between the PCOS and control groups demonstrate the role of DEp53TGs in promoting apoptosis, cellular senescence, and cell cycle arrest, while simultaneously reducing the proliferation of GCs [25]. Furthermore, research indicates that MDM2 expression levels are lower in human granulosa cells, leading to decreased oocyte maturation and fertilization rates [116]. This phenomenon is commonly observed in women with PCOS, suggesting that the MDM2-p53 axis in ovarian granulosa cells holds significant clinical relevance for human fertility [116]. This process is thought to primarily affect the stages from small antral follicles to dominant follicle selection, thereby interfering with normal follicular fate determination. In contrast, direct evidence for p53-mediated ferroptosis in PCOS remains limited, derived mainly from animal models and indirect inferences. Existing studies suggest that hyperandrogenism can downregulate hepcidin expression, leading to increased iron loading in ovarian tissues; in this context, the inhibition of SLC7A11 by p53 may synergize with iron overload to reduce GPX4 activity and promote the accumulation of lipid peroxides [117]. This parallel shift, characterized by enhanced apoptosis and increased susceptibility to ferroptosis, may collectively contribute to follicular development arrest and the progressive impairment of ovarian reserve, although further validation in human PCOS ovaries is still required. In terms of therapeutic exploration, targeted modulation of p53 activity (e.g., via the MDM2 inhibitor Nutlin-3a) or inhibition of the ferroptotic process (e.g., using Liproxstatin-1) has demonstrated potential in PCOS animal models to improve the oxidative status of ovarian tissues and restore follicular structure [118]. However, these strategies currently remain in the experimental stage, and their safety, ovarian specificity, and impact on reproductive outcomes in PCOS still require systematic evaluation.

#### Mitochondria: integration platform for apoptosis and ferroptosis

As the central organelles for intracellular energy metabolism and redox regulation, mitochondria are considered to play an integrative role in orchestrating signaling pathways related to both apoptosis and ferroptosis under specific pathological stress conditions. In apoptosis, mitochondrial outer membrane permeabilization (MOMP) leads to cytochrome c release into the cytosol, activating the caspase-9/3 cascade to execute programmed cell death [119, 120]. In ferroptosis, mitochondria undergo characteristic morphological changes, including cristae loss, increased membrane density, and outer membrane shrinkage, along with the accumulation of high levels of lipid peroxidation products (4-HNE). These pathways are interconnected through iron metabolism. In ferroptosis, labile Fe²⁺ drives ROS generation via the Fenton reaction, inducing lipid peroxidation [121]. Meanwhile, apoptosis-activated caspase-3 cleaves the iron exporter FPN1 (ferroportin-1), thereby restricting iron efflux and expanding the labile iron pool (LIP) [122]. This mechanism suggests that, under specific conditions, apoptosis activation may enhance cellular sensitivity to ferroptosis; however, its prevalence in ovarian cells remains to be further validated. Consequently, mitochondria are regarded as a vital structural and biochemical platform where apoptosis and ferroptosis signaling pathways likely converge.

In PCOS, hyperandrogenic microenvironments disrupt mitochondrial homeostasis, synergistically driving dual death programs in granulosa cells. Clinical observations suggest pronounced mitochondrial structural and iron metabolism abnormalities in PCOS granulosa cells: reduced mitochondrial volume, cristae loss, outer membrane rupture, and elevated lipid peroxidation marker 4-HNE [12, 123]. As a terminal lipid peroxidation product, 4-HNE can upregulate Fas expression, activate caspase-3, and ultimately induce apoptosis [57]. This multidimensional dysregulation activates death pathways through dual mechanisms: inducing MOMP to increase caspase-3 activity and initiate the apoptotic cascade, while simultaneously reducing GPX4 activity to promote ferroptosis. Mechanistically, hyperandrogenism exacerbates pathology via two synergistic effects: (1) inducing overexpression of dynamin-related protein 1 (DRP1), disrupting mitochondrial dynamics and increasing MOMP susceptibility; (2) downregulating hepcidin, causing iron overload and amplifying ROS bursts via Fenton reactions. Ultimately, this results in massive granulosa cell loss, accelerated follicular atresia, and progressive decline of ovarian reserve function [124, 125].

### Ferroptosis–pyroptosis crosstalk

#### Reactive Oxygen Species (ROS) storm

Reactive oxygen species (ROS) are considered critical amplification factors linking ferroptosis and pyroptosis under various stress conditions, facilitating their mutual reinforcement within specific pathological environments. On one hand, pyroptotic execution markedly enhances ROS generation. Activation of inflammasomes such as NLRP3 induces Gasdermin D (GSDMD) to form plasma membrane pores. This process damages mitochondria, leading to loss of membrane potential and respiratory chain abnormalities, which in turn release large amounts of mitochondrial ROS (mtROS) [126]. Inflammasome signaling can also activate NADPH oxidases (NOX), further exacerbating intracellular oxidative stress [127]. The sustained elevation of ROS can directly attack membrane phospholipids enriched with polyunsaturated fatty acids (PUFA-PLs), accelerating the chain reaction of lipid peroxidation (LPO). This process depletes glutathione (GSH) and inhibits the activity of glutathione peroxidase 4 (GPX4), thereby increasing cellular susceptibility to ferroptosis [121].

On the other hand, ferroptosis itself is a potent ROS generator. Its core features—iron-dependent Fenton reactions and lipoxygenase (LOX) activity—may lead to the accumulation of LPO products (lipid hydroperoxides L-OOH) and secondary oxidized products (reactive aldehydes) [128]. These molecules not only directly damage cell membranes, causing rupture, but also act as strong damage-associated molecular patterns (DAMPs) and endogenous danger signals. They can be recognized by pattern recognition receptors (PRRs, TLRs, RAGE) or serve as a “Signal 2” to activate NLRP3 inflammasome assembly, thereby triggering GSDMD-mediated pyroptosis [129]. Experimental data suggest that ROS may form a positive feedback loop between ferroptosis and pyroptosis.During pyroptosis, inflammasome activation during pyroptosis elevates ROS levels.The increased ROS—particularly mitochondrial ROS—accelerates lipid peroxidation and impairs antioxidant defenses through GSH depletion and GPX4 inhibition, thereby promoting ferroptosis. Ferroptotic cell rupture, in turn, releases DAMPs and endogenous danger signals such as lipid peroxidation products, HMGB1, and ATP. These molecules activate pattern-recognition receptors (e.g., TLRs, NLRP3 inflammasome) in neighboring cells, triggering additional pyroptosis and further amplifying ROS production. This self-reinforcing cycle may serve as a central hub contributing to cascading cell death and progressive tissue inflammation.

In the context of PCOS, a complex reproductive–endocrine–metabolic disorder, ROS-mediated ferroptosis–pyroptosis crosstalk likely contributes to key pathophysiological processes. PCOS patients commonly exhibit chronic low-grade inflammation, hyperandrogenism, and insulin resistance, collectively promoting sustained oxidative stress and excessive ROS accumulation [130]. Hyperandrogenism (testosterone) stimulates ovarian theca and stromal cells to produce excess ROS; insulin resistance and hyperinsulinemia exacerbate oxidative damage by activating NADPH oxidases and impairing mitochondrial function [131]. This persistent ROS environment provides the pathological basis for ferroptosis–pyroptosis crosstalk.Locally, ROS surges in the ovary directly induce lipid peroxidation damage in granulosa cells, theca cells, and luteal cells, driving ferroptosis [12]. Concurrently, ROS and ROS-mediated lipid peroxidation products (MDA, 4-HNE) act as endogenous DAMPs. In combination with elevated circulating inflammatory cytokines in PCOS (TNF-α, IL-1β, IL-18), these signals continuously activate NLRP3 inflammasomes in ovarian macrophages and stromal cells, triggering GSDMD-dependent pyroptosis [132]. Mature pro-inflammatory cytokines (IL-1β, IL-18) and newly released DAMPs further amplify local oxidative stress and promote insulin resistance, forming a self-reinforcing pathological loop. The crosstalk between these death pathways results in multiple pathological consequences: granulosa cell loss causing follicular arrest, ovulatory dysfunction, luteal structural abnormalities, and progressive ovarian stromal fibrosis [133, 134]. Moreover, this mechanism may extend beyond the ovary, contributing to endometrial damage, adipose tissue and hepatic metabolic inflammation, and systemic insulin-target organ dysfunction [135–137]. Therapeutic strategies targeting ROS homeostasis, inhibiting NLRP3 inflammasome activation, enhancing Nrf2-mediated antioxidant defense, or modulating iron metabolism hold potential for ameliorating ovarian dysfunction and metabolic complications in PCOS.

#### Mutual amplification of inflammatory signaling

As a highly inflammatory form of programmed cell death, pyroptotic cells release a variety of pro-inflammatory cytokines (such as interleukin-1β [IL-1β] and interleukin-18 [IL-18]) and damage-associated molecular patterns (DAMPs), including high mobility group box 1 (HMGB1) and ATP [138]. These molecules can remodel the local tissue microenvironment through autocrine or paracrine signaling [139]. At the transcriptional level, in vitro and animal studies have demonstrated that these inflammatory mediators can modulate the expression of ferroptosis-related gene networks—for instance, by upregulating the pro-lipid peroxidative acyl-CoA synthetase long-chain family member 4 (ACSL4) and transferrin receptor (TfR), while suppressing key antioxidant defense molecules, including the cystine/glutamate antiporter System Xc − and glutathione peroxidase 4 (GPX4) [140, 141]. Additionally, these mediators recruit and activate immune effector cells such as macrophages and neutrophils, promoting the release of ROS bursts, tissue proteases, and tumor necrosis factor-α (TNF-α), collectively creating a pro-oxidative environment that favors ferroptosis [142]. Notably, Gasdermin-mediated plasma membrane pores not only disrupt ion homeostasis but also facilitate the leakage of intracellular antioxidants (glutathione, ubiquinone), directly compromising the cellular antioxidant defense system [143]. Conversely, the crosstalk from ferroptosis to pyroptosis relies on the dual drivers of danger signal transmission and organelle damage. Upon ferroptotic cell rupture, characteristic DAMPs—including HMGB1, mitochondrial DNA, ATP, and lipid peroxidation products—are specifically recognized by pattern recognition receptors (PRRs) on neighboring macrophages, activating NF-κB signaling to upregulate basal expression of inflammasome components [144]. Sim"ltaneous"y, accumulated lipid peroxides and their secondary metabolites act as a “signal 2” to trigger NLRP3 inflammasome assembly and activation [145]. Importantly, ferroptosis-induced mitochondrial ultrastructural changes—such as cristae collapse and membrane depolarization—accompany explosive mtROS release, serving as potent inflammasome activators [146].

In the context of PCOS, this ferroptosis–pyroptosis crosstalk exhibits pronounced tissue-specific pathological effects under a chronic inflammatory and metabolic dysregulation microenvironment. Within the ovary, hyperandrogenism combined with insulin resistance drives ROS bursts that preferentially induce lipid peroxidation in granulosa cells, initiating ferroptosis [63, 147]. Meanwhile, the accumulation of inflammatory cytokines (IL-1β, TNF-α) and lipid peroxidation markers (such as 4-HNE and MDA) in the follicular fluid can activate the NLRP3 inflammasome in ovary-resident macrophages and induce pyroptosis in granulosa cells through paracrine signaling [147]. The synergistic activation of these bidirectional death pathways is thought to primarily impact the transition from small antral follicles to dominant follicles, leading to the progressive loss of granulosa cells, follicular development arrest, ovulatory dysfunction, and abnormalities in luteal-related functions. Meanwhile, persistent inflammatory stimulation may accelerate ovarian stromal fibrosis [148]. Notably, this ferroptosis–pyroptosis crosstalk is not strictly confined to ovarian tissues. For instance, under conditions of cyclic oxidative stress exposure, activation of ferroptosis- and pyroptosis-related signaling has also been observed in endometrial cells, which may impair endometrial receptivity and further exacerbate adverse reproductive outcomes [149]. However, the specific contribution of this expanded mechanism in PCOS patients remains to be validated by more direct evidence.

### Metabolic death pathway crosstalk: ferroptosis–autophagy interaction network

#### Ferritinophagy (Iron-Selective Autophagy)

Ferritinophagy is a selective autophagic process that regulates intracellular iron homeostasis. Its molecular mechanism relies on the autophagy receptor NCOA4, which specifically recognizes and binds ferritin complexes [150]. NCOA4 targets ferritin to autolysosomes for degradation, releasing large amounts of labile ferrous iron (Fe²⁺) into the cytoplasm [151]. The liberated Fe²⁺ catalyzes the production of reactive oxygen species (ROS) via Fenton reactions, driving uncontrolled lipid peroxidation (LPO) of polyunsaturated phospholipids (PUFA-PLs) and ultimately triggering ferroptosis [152].

In PCOS, patients often exhibit elevated serum ferritin and local ovarian iron overload, indicating that dysregulated iron metabolism contributes to disease progression. Within the PCOS-specific ovarian microenvironment, hyperandrogenism and insulin resistance synergistically activate autophagy signaling, enhancing NCOA4-mediated ferritinophagy in granulosa cells [153]. This process results in sustained cytoplasmic Fe²⁺ accumulation, catalyzing a lipid peroxidation cascade via Fenton chemistry and markedly exacerbating oxidative stress, directly damaging the lipid membranes of follicular fluid and granulosa cells [154]. Furthermore, some studies suggest that ferritinophagy may interact with the chaperone-mediated autophagy (CMA) pathway to promote the degradation of the key antioxidant enzyme GPX4 under specific conditions, thereby further weakening the cell’s capacity to scavenge lipid reactive oxygen species (lipid ROS) [155]. In this context, enhanced ferritinophagy may increase the susceptibility of granulosa cells to ferroptosis, rather than acting as a sufficient trigger on its own. Ferroptosis-related membrane disruption is thought to accelerate granulosa cell loss and primarily affect the transition from small antral follicles to dominant follicles, thus promoting follicular atresia, ovulatory dysfunction, and the progressive decline of ovarian reserve [153]. Evidence supporting this view primarily stems from animal model studies. For instance, in PCOS animal models, NCOA4 expression levels have been observed to correlate positively with the accumulation of lipid peroxidation markers, such as 4-hydroxynonenal (4-HNE), in ovarian tissues [153]. Although these findings suggest that the ferritinophagy–ferroptosis axis may be involved in the pathogenesis and progression of PCOS-related ovarian dysfunction, its specific regulatory mechanisms and causal relationships in the human ovary remain to be further validated. Consequently, this pathway is currently better regarded as a potential pathological mechanism and an exploratory therapeutic target, rather than an established primary pathogenic axis.

#### Degradation of antioxidant defense (GPX4)

Chaperone-mediated autophagy (CMA) regulates ferroptosis sensitivity through selective degradation of the key antioxidant enzyme glutathione peroxidase 4 (GPX4) [156]. This process is initiated by the heat shock protein HSP90, which recognizes cytosolic GPX4 and forms a complex that is subsequently targeted to the lysosomal membrane receptor LAMP2A. Under the mediation of LAMP2A, GPX4 is translocated into the lysosomal lumen and degraded by proteases, resulting in a marked reduction of cellular GPX4 protein levels [157]. As a critical enzyme repairing phospholipid hydroperoxides, GPX4 loss leads to irreversible accumulation of lipid peroxides, such as phosphatidylethanolamine–polyunsaturated fatty acid adducts, thereby disrupting membrane integrity and redox homeostasis, ultimately increasing cellular susceptibility to ferroptosis [158]. Experimental evidence supports that CMA-specific inhibitors or HSP90 antagonists effectively block GPX4 degradation, restore antioxidant capacity, and suppress ferroptotic cell death [159].

In the pathological context of PCOS, hyperandrogenism and chronic inflammation–induced oxidative stress abnormally activate the CMA pathway [160]. Animal models and limited clinical observations have shown that LAMP2A expression levels are upregulated in the ovarian granulosa cells of PCOS patients and negatively correlate with GPX4 protein expression. This suggests that CMA-mediated GPX4 degradation may be involved in the formation of ovarian oxidative damage [161]. Under sustained ROS generation in a hyperandrogenic environment, the HSP90–LAMP2A axis accelerates lysosomal degradation of GPX4, compromising cellular capacity to detoxify lipid peroxides. This effect synergizes with ferritinophagy-derived labile Fe²⁺, amplifying the lipid peroxidation “storm” and promoting ferroptotic death of granulosa cells [162]. In this context, the continuous loss of granulosa cells may disrupt follicular microenvironmental homeostasis, interfere with steroidogenesis, and promote follicular atresia, thereby correlating with the phenotypes of ovulatory dysfunction and impaired ovarian reserve in PCOS. Notably, although existing evidence supports the critical role of the CMA–GPX4 axis in regulating oxidative stress and ferroptosis, its spatio-temporal specific regulation and causal relationship in the PCOS ovary remain largely inferred from experimental models and require more direct validation at the human tissue level. This mechanism offers a novel molecular perspective for understanding the link between oxidative stress and ferroptosis in PCOS and lays the foundation for subsequent mechanism-oriented research.

#### Lipid metabolism: lipophagy

Lipophagy, a selective form of autophagy, dynamically regulates intracellular lipid metabolism by degrading lipid droplets (LDs). This process involves autophagosomal sequestration of neutral lipid cores, followed by lysosomal hydrolysis via lysosomal acid lipase (LAL), releasing free fatty acids (FFAs) [163]. Polyunsaturated fatty acids (PUFAs), such as arachidonic acid (AA) and adrenic acid (AdA), are subsequently activated by acyl-CoA synthetase long-chain family member 4 (ACSL4) and esterified into phosphatidylethanolamine-PUFA (PE-PUFA), which serve as central substrates for lipid peroxidation. In the presence of labile Fe²⁺, PE-PUFAs undergo Fenton reaction-mediated lipid radical (L-ROS) formation, directly driving the execution of ferroptosis [164]. Genetic inhibition of lipophagy (ATG7 knockout) reduces PUFA release and confers significant resistance to ferroptosis, whereas pharmacological activation of lipophagy with rapamycin enhances lipid droplet clearance and increases ferroptotic sensitivity [165]. These findings indicate that lipophagy plays a significant role as an amplifier in the regulation of ferroptosis by modulating the accessibility of lipid peroxidation substrates.

In PCOS, lipophagy contributes to metabolic dysregulation via tissue-specific mechanisms. Adipose tissue exhibits hyperactive lipophagy, wherein insulin resistance relieves mTORC1-mediated suppression of autophagy, leading to excessive lipid droplet degradation and overproduction of circulating FFAs enriched in PUFAs [49]. These FFAs not only induce lipotoxicity in peripheral organs such as liver and muscle but also provide key substrates for lipid peroxidation in the ovarian microenvironment. Ovarian granulosa cells undergo lipid metabolic reprogramming: hyperandrogenism activates androgen receptor (AR) signaling, upregulating fatty acid–binding protein 4 (FABP4) to facilitate PUFA uptake. Meanwhile, androgen-associated autophagy activation may accelerate the degradation of intracellular lipid droplets. The released PUFAs are then esterified by ACSL4 to form PE-PUFAs, mechanistically providing the necessary conditions for the occurrence of lipid peroxidation and ferroptosis [35]. It should be noted that evidence regarding the direct role of lipophagy in driving ferroptosis in PCOS granulosa cells currently stems primarily from indirect inferences based on research into metabolic abnormalities and ferroptotic mechanisms. The causal relationship remains to be confirmed by further experiments targeting human PCOS ovarian tissues and cellular models. Nevertheless, existing studies collectively suggest that the lipophagy–PUFA–lipid peroxidation axis may play a promotive role in PCOS-related follicular atresia and ovarian dysfunction.

#### Mitophagy: dual role

Mitophagy, as a selective mechanism for removing damaged or surplus mitochondria, serves as a key quality control process and exhibits a complex, context-dependent dual role in regulating ferroptosis. On one hand, moderate mitophagy has the potential to suppress ferroptosis: by timely eliminating dysfunctional, ROS-generating mitochondria, it effectively alleviates intracellular oxidative stress, thereby reducing the risk of lipid peroxidation-driven ferroptosis [166]. On the other hand, under specific stress conditions, abnormally enhanced or persistently activated mitophagy may be associated with an increased susceptibility to ferroptosis [167]. This promotive effect is primarily realized through two interconnected pathways: First, excessive clearance of mitochondria may lead to compromised cellular energy homeostasis. A significant decline in mitochondrial number and function impairs oxidative phosphorylation, resulting in insufficient ATP production. This ATP depletion, in turn, disrupts the biosynthesis of glutathione (GSH), which provides the reducing power necessary for maintaining redox homeostasis and the activity of glutathione peroxidase 4 (GPX4)—a key ferroptosis inhibitor [168]. Second, mitochondria serve as vital functional platforms for various antioxidant defense systems. Mitochondrial dysfunction or excessive clearance may weaken the overall cellular antioxidant buffering capacity, rendering the cell more susceptible to oxidative damage under high iron or lipid peroxidation stress [169]. Consequently, the role of mitophagy in regulating ferroptosis is non-linear, depending on the dynamic equilibrium between ‘moderate protection’ and ‘decompensatory imbalance’.

In PCOS, patients commonly exhibit granulosa cell dysfunction, aberrant follicular development, and metabolic disturbances. Studies have shown that mitochondrial dysfunction and oxidative stress are significantly elevated in the ovarian microenvironment of PCOS patients [170]. Under these conditions of sustained stress, mitochondrial damage and excessive ROS production may stimulate mitophagy as a compensatory protective response [171]. Theoretically, moderate mitophagy should facilitate the clearance of damaged mitochondria and alleviate oxidative stress, thereby maintaining granulosa cell survival. However, several studies simultaneously suggest that in PCOS, overall autophagic flux is impaired or the efficiency of selective mitophagy is diminished. This may lead to the ineffective clearance of damaged mitochondria, triggering persistent ROS accumulation and enhanced lipid peroxidation, which increases the susceptibility of granulosa cells to ferroptosis—a process that potentially accelerates follicular atresia [172]. On the other hand, in certain PCOS subtypes or pathological stages, overactivation of mitophagy may also occur, particularly under severe energy stress or abnormal activation of specific signaling pathways. This may lead to ATP depletion and loss of mitochondrial antioxidant reserves as described above, weakening cell survival and potentially promoting ferroptosis in key cell types, further impairing follicular development and ovarian function. Moreover, insulin resistance, commonly observed in PCOS, may interfere with autophagy regulation (including mitophagy) via the AMPK/mTOR pathway, forming a vicious cycle [50]. Importantly, the proposed death crosstalk network should be regarded as a conceptual integrative framework rather than a fully validated ovarian signaling hierarchy. While emerging evidence supports multiple points of interaction among regulated cell death pathways, most mechanistic links in PCOS remain inferred from animal models or non-ovarian systems, underscoring the need for stage- and cell-specific validation in human ovarian tissues.

**Fig. 2:**
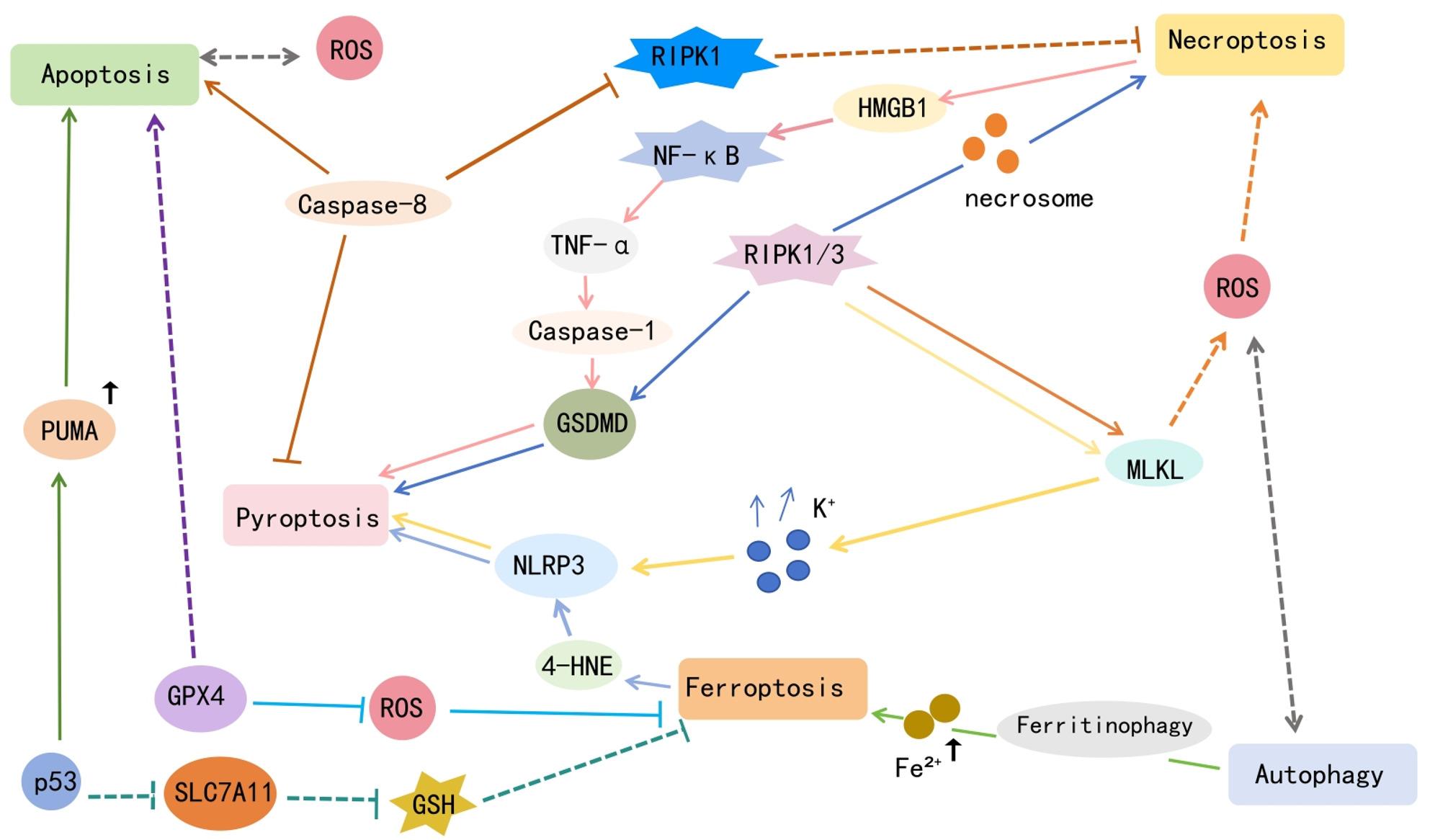
Conceptual framework of the interconnected death-crosstalk network in PCOS

To more clearly integrate the programmed cell death pathways described above, this review further summarizes how key PCOS-associated stressors—including reactive oxygen species (ROS), inflammatory mediators, and perturbations in iron homeostasis—may drive the cross-activation of multiple death programs within the follicular microenvironment. As illustrated in Fig. 2, central regulatory nodes such as RIPK1/3, NF-κB, TNF-α, ROS, and iron-dependent lipid peroxidation potentially link apoptosis, pyroptosis, necroptosis, ferroptosis, and autophagy, forming a dynamic and mutually reinforcing death-crosstalk network. This integrative schematic complements the pathway-specific discussions above and provides a systems-level framework for understanding the molecular basis of follicular dysfunction in PCOS, primarily based on experimental and preclinical evidence. 

This schematic illustrates how PCOS-specific stressors (e.g., hyperandrogenism and insulin resistance) converge on key molecular hubs to orchestrate multiple programmed cell death (PCD) pathways. (A) Experimentally Validated Links in PCOS: Solid arrows represent interactions with direct evidence in PCOS models (human granulosa cells or animal models), including ROS-driven NLRP3 activation, lipid peroxidation-mediated ferroptosis, and caspase-dependent apoptosis. (B) Mechanistic Crosstalk Hubs: RIPK1, TNF-α, and Caspase-8 serve as central switches linking apoptosis, pyroptosis, and necroptosis. While these pathways are well-established in general cell biology, their integrated role in PCOS ovaries is proposed based on shared signaling mediators (e.g., GSDMD, MLKL). (C) Hypothesis-Driven Extrapolations: Dashed lines indicate potential feedback loops, such as ferritinophagy-driven ferroptosis and MLKL-associated ROS amplification, which represent theoretical integration points for future PCOS research. Overall, the model suggests that follicular impairment in PCOS may result from a dynamic network rather than isolated death pathways, providing a roadmap for multi-target therapeutic intervention.

## Cell death interconnection networks in PCOS: mechanistic insights and translational perspectives

In this section, we discuss the potential of modulating cell death networks as a strategy to restore ovarian function. However, it is important to clarify that while certain modulators—such as RIPK1 inhibitors, ferroptosis quenchers, and YAP inhibitors—have provided pivotal mechanistic insights in laboratory settings, their transition to clinical practice remains purely speculative. Most of these findings are derived from non-ovarian models or preclinical PCOS rodents, and their safety and efficacy in human reproductive health have yet to be established.

### Repurposed metabolic and anti-inflammatory agents modulating death crosstalk

Accordingly, the therapeutic strategies discussed in this section are intended primarily to illustrate how key death-signaling nodes within the crosstalk network may be experimentally interrogated or conceptually targeted. Rather than proposing immediate clinical interventions, these examples highlight mechanistic points of convergence between metabolism, inflammation, and regulated cell death that may inform future, stage- and tissue-specific therapeutic development in PCOS.

#### Metformin: multi-pathway regulatory roles driven by metabolic stress hubs

As a classic insulin sensitizer, metformin primarily improves PCOS-related metabolic disorders by activating the AMP-activated protein kinase (AMPK) signaling pathway [26]. Recent studies suggest that its therapeutic benefits may partially involve the modulation of an ovarian stress-response network synergistically driven by hyperandrogenism and insulin resistance, rather than directly targeting a single cell death pathway. Mechanistically, metformin induces AMPK phosphorylation, inhibits androgen-related inflammatory signal transduction, and has been observed in vitro and in animal models to attenuate NLRP3 inflammasome activity, thereby reducing the propensity for downstream pro-inflammatory effects (including GSDMD-related events) [173]. Simultaneously, metformin can enhance mitochondrial quality control through the ETHE1/KEAP1/NRF2 axis and promote mitophagy to clear damaged mitochondria—characterized by mitochondrial reactive oxygen species (mtROS) accumulation and cristae disruption—thus alleviating the cellular oxidative stress load [174]. In preclinical models, this AMPK-centered metabolic reprogramming and anti-inflammatory effect are thought to indirectly influence the activation thresholds of various regulated cell death processes by reducing the overall cellular susceptibility to oxidative damage and lipid peroxidation stress, rather than specifically intervening in a single mode of death [175]. At the clinical level, metformin treatment has been proven to improve the metabolic status of PCOS patients, reduce circulating levels of inflammatory cytokines, and alleviate ovarian stromal fibrosis as well as markers associated with oxidative damage [176]. However, explicit evidence remains lacking as to whether it directly regulates the switching of cell death modes in human ovarian granulosa cells. Furthermore, in the context of combined lifestyle interventions, metformin may further weaken the long-range impact of lipid excess on ovarian oxidative stress and cellular damage by improving hepatic lipid metabolic homeostasis and reducing the systemic load of circulating free fatty acids [177].

#### Multi-target intervention mechanisms of anti-inflammatory drugs

Glucagon-like peptide-1 receptor agonists (GLP-1RAs) (liraglutide) inhibit NF-κB nuclear translocation associated with insulin resistance in PCOS, thereby blocking the TNF-α/RIPK1-mediated necroptotic signaling cascade and significantly suppressing MLKL phosphorylation and membrane translocation. This process directly counteracts the dysregulated death-switch equilibrium within the hyperandrogenic microenvironment, reducing the release of damage-associated molecular patterns (DAMPs) and consequently alleviating TGF-β1-driven ovarian inflammatory fibrosis [178, 179]. Natural anti-inflammatory compounds (such as curcumin) can likewise participate in the pathological improvement of PCOS through multi-level regulation of oxidative stress and inflammatory responses. Their ability to scavenge reactive oxygen species (ROS) helps reduce the oxidative stress load. In various preclinical models, they have been reported to inhibit the DAMP–TLR4 signaling axis and the activation of the NLRP3 inflammasome, thereby decreasing the propensity for inflammation-related cellular damage and GSDMD-related events [180]. Furthermore, curcumin has been found to enhance the functionality of antioxidant defense systems, maintaining glutathione peroxidase 4 (GPX4) activity to a certain extent and alleviating lipid peroxidation damage. However, the specific role of these effects in the regulation of ovarian-specific cell death remains to be further validated. Notably, some studies suggest that lipid peroxidation products, such as 4-hydroxynonenal (4-HNE), may amplify the crosstalk between inflammation and cell damage signals by covalently modifying protein cysteine residues [181]. Anti-inflammatory and antioxidant interventions, by reducing 4-HNE accumulation, may indirectly weaken the positive feedback amplification loop between iron-dependent lipid peroxidation and inflammatory cell death. However, the specific molecular targets of this process in PCOS ovarian tissue remain to be further validated [94]. Overall, by collectively modulating the ‘inflammation–oxidative stress–cellular damage’ core hub in PCOS, GLP-1RAs and natural anti-inflammatory compounds may provide indirect support for improving ovarian microenvironmental abnormalities and ovulatory dysfunction. However, the specific functional pathways remain to be elucidated through more refined mechanistic studies.

### Emerging experimental modulators of death-signaling hubs

It should be emphasized that the following strategies are discussed primarily as experimental tools to interrogate regulated cell death crosstalk and to validate causal death nodes in PCOS, rather than as immediately translatable therapeutic approaches.

#### NLRP3 inflammasome inhibitors: targeting the hub for inflammation-driven death signal amplification

In the chronic inflammatory microenvironment of PCOS, the NLRP3 inflammasome is considered a vital signal amplification hub connecting inflammatory responses with various forms of programmed cell death. Inhibitory strategies targeting NLRP3 primarily intervene in the assembly process of the inflammasome to inhibit caspase-1 activation. This reduces GSDMD cleavage as well as the maturation and release of IL-1β and IL-18, thereby limiting the sustained amplification of inflammatory cell damage [182]. Various natural or synthetic compounds have been reported to possess the potential to inhibit NLRP3 inflammasome activation. For instance, resveratrol can inhibit the oligomerization and downstream signaling of the NLRP3 inflammasome by modulating its conformational changes and energy-dependent processes [183]. Meanwhile, the highly selective NLRP3 inhibitor MCC950 and its derivatives have been shown to block ASC speck formation, effectively suppressing inflammasome activation [184]. In PCOS animal models, such interventions can alleviate ovarian inflammation and are correlated, to some extent, with improvements in reproductive endocrine parameters and ovulatory phenotypes [185]. However, the causal mechanisms and cell-type-specific roles remain to be further elucidated. Beyond directly influencing the process of inflammatory cell damage, the inhibition of the NLRP3 inflammasome may exert synergistic regulatory effects across various cell death signaling pathways. On one hand, the reduction in inflammasome activity can decrease disturbances in cellular ionic homeostasis and the release of damage-associated molecular patterns (DAMPs), thereby indirectly weakening the amplification of inflammatory pathways by necroptosis-related signaling [186]; on the other hand, in the context of combined strategies with ferroptosis inhibitors, NLRP3 inhibition may interfere with the positive feedback amplification loop between iron-dependent lipid damage and inflammatory cell death by reducing the reactivation of inflammasomes induced by lipid peroxidation products [187]. Consequently, intervention strategies targeting the NLRP3 inflammasome provide a conceptual framework for understanding and remodeling the inflammation-driven cell death signaling network in PCOS. These strategies hold promise for restoring ovarian microenvironmental homeostasis and exploring the potential of multi-pathway synergistic regulation. However, the precise mechanisms of action across different PCOS phenotypes and key ovarian cell types still require validation through systematic mechanistic studies and more targeted experimental models.

#### RIPK1/RIPK3 pathway inhibitors: intervening in the pathological switch between apoptosis and necroptosis

Targeting the RIPK1/RIPK3 signaling axis is considered a strategy with theoretical potential for intervening in the abnormal switching of cell death modes in PCOS, particularly in pathological scenarios characterized by an inflammation-driven imbalance between apoptosis and necroptosis (see sections "Central hub role of the RIPK1–RIPK3–MLKL axis"-"Death decision switch function of caspase-8"). In an ovarian microenvironment where hyperandrogenism and chronic inflammation coexist, biased death receptor signaling and imbalanced caspase activity may drive the transition of RIPK1 from a pro-apoptotic regulator to a signaling node for necroptosis. In experimental models, the RIPK1 kinase activity inhibitor Necrostatin-1 (primarily used as a research tool) can interfere with RIPK1-dependent necroptotic signaling by inhibiting the autophosphorylation of its kinase domain, thereby limiting the abnormal downstream activation of MLKL [188]. Simultaneously, RIPK3-dependent necrosome assembly (mediated by the RHIM domain) can be antagonized by selective inhibitors such as GSK’872, which subsequently inhibits MLKL oligomerization and its translocation to the plasma membrane [189]. The aforementioned processes are closely linked to the disruption of plasma membrane permeability and the release of damage-associated molecular patterns (DAMPs, such as HMGB1), which are thought to amplify local inflammation and tissue damage signals. By limiting the release of necroptosis-related DAMPs, the inhibition of the RIPK1/RIPK3 pathway may also indirectly weaken the inflammatory cascade mediated by the DAMPs–TLR4–NF-κB axis, thereby influencing the inflammatory phenotypic bias (such as M1 polarization) of local ovarian macrophages [190]. This effect may contribute to alleviating TGF-β1-driven abnormal collagen deposition and ovarian stromal remodeling, and has been observed in animal models alongside improvements in ovarian structural parameters and ovulatory phenotypes. However, the specific causal relationship and cell-type-specific mechanisms remain to be further clarified [191].Although current evidence is primarily derived from experimental models, the RIPK1/RIPK3 signaling axis—as a critical node connecting cell death mode switching and inflammatory amplification—provides an important theoretical framework for understanding the interaction between inflammation and cell death in the pathological progression of PCOS, rather than serving as an established clinical intervention strategy.

#### Intervention strategies targeting the ferroptosis–pyroptosis crosstalk

Intervention strategies targeting ferroptosis aim to act on cell death interaction nodes with signal amplification effects within the PCOS ovarian microenvironment by synergistically regulating lipid peroxidation and inflammatory cascades [62]. The iron chelator deferoxamine can sequester abnormally accumulated free iron in the hyperandrogenic microenvironment, limiting the Fenton reaction-driven lipid peroxidation cascade and thereby reducing the risk of ferroptosis initiation [192]. Furthermore, by decreasing the generation of lipid peroxidation products (such as 4-hydroxy-2-nonenal, 4-HNE), such interventions are thought to indirectly weaken the modification of key cysteine residues of GSDMD (e.g., Cys191) by 4-HNE, thus reducing the propensity for events associated with pyroptotic pore formation [193]. Notably, lipid peroxidation products have been proposed to participate in the development of receptor dysfunction in ovarian granulosa cells. Although direct evidence remains limited, reducing 4-HNE load could theoretically help alleviate oxidative stress-mediated modifications of receptor proteins, thereby partially restoring the sensitivity of granulosa cells to follicle-stimulating hormone (FSH) signaling. However, this inference warrants further validation in ovary-specific models [193, 194]. Simultaneously, strategies to enhance intracellular anti-lipid peroxidation capacity have been employed to explore the regulatory mechanisms of ferroptosis. For instance, potentiation of GPX4 function (achieved through GPX4 activators or epigenetic regulatory pathways in various experimental systems, such as the YTHDF1/SLC7A5 axis) can upregulate glutathione peroxidase 4 (GPX4) expression and enhance the cellular capacity to scavenge lipid peroxides, thereby inhibiting the occurrence of ferroptosis [195]. Furthermore, the reduction in lipid peroxidation levels and the alleviation of mitochondrial reactive oxygen species (mtROS) are thought to limit the aberrant activation of the NLRP3 inflammasome, indirectly weakening the GSDMD-dependent pyroptotic execution process [196]. Taken together, by simultaneously intervening in the lipid peroxidation process and the inflammasome-driven pyroptotic amplification loop, this strategy theoretically helps interrupt the positive feedback loop between ferroptosis and pyroptosis. Although current evidence is primarily derived from experimental studies, this approach provides a conceptual framework for understanding the cross-regulation of cell death pathways in PCOS and serves as a reference for future explorations of synergistic regulatory mechanisms.

#### YAP inhibitors (Innovative Target)

Given the critical regulatory role of the Yes-associated protein (YAP) signaling pathway in integrating cellular mechanical response, inflammatory reactions, and metabolic reprogramming, its potential involvement in hyperandrogenism and inflammation-related cell death in PCOS has increasingly garnered attention. Existing research suggests that YAP signaling may serve as a bridge coordinating hyperandrogen exposure, NF-κB inflammatory activation, and pyroptotic responses, making it an intervention target of significant exploratory value [32]. Recent experimental studies have focused on the application of the YAP inhibitor verteporfin, which is reported to interfere with the function of the YAP–transcription factor complex, thereby inhibiting the abnormal activation of the YAP–NF-κB signaling axis under hyperandrogenic conditions [197]. By suppressing this pathway, verteporfin has been observed to reduce the assembly efficiency of the downstream NLRP3 inflammasome and attenuate the Gasdermin D (GSDMD)-mediated pyroptotic execution process [32, 198]. This pronounced inhibition of the NLRP3/GSDMD pyroptotic pathway produces clear therapeutic effects in PCOS experimental models: it not only significantly reduces abnormal accumulation of cystic follicles in the ovary but also markedly decreases the release of key pro-inflammatory cytokine IL-1β [32]. These observations support the hypothesis that, within the context of hyperandrogen exposure, the YAP–NF-κB–NLRP3/GSDMD signaling axis may participate in the amplification of ovarian inflammation and the regulation of cellular damage. However, its specific mechanisms of action across different ovarian cell types remain to be further elucidated.Although current evidence is primarily derived from experimental studies, and the cell-type specificity and long-term safety of YAP signaling regulation are not yet clear, this research direction provides a novel conceptual perspective for understanding the coupling between mechanical signaling, inflammatory response, and pyroptotic regulation in PCOS. It further suggests that YAP may serve as a critical regulatory node in the inflammation–cell death interaction network, warranting further investigation.

### Genetic and epigenetic regulators as mechanistic probes of cell death dysregulation

#### miRNA-targeted therapy

Given that microRNAs (miRNAs) play a crucial role in the fine-tuning of various cell fate-related signaling pathways at the post-transcriptional level, their potential involvement in the abnormal programmed cell death regulatory network in PCOS has increasingly garnered attention. Recent basic and translational studies suggest that specific miRNAs may be associated with granulosa cell dysfunction and homeostatic imbalance by influencing processes such as autophagy, apoptosis, and ferroptosis. In experimental research, interventions targeting miR-125b have been employed to explore its role in the regulation of granulosa cell autophagy [199]. For instance, anti-miR-125b-based delivery systems have been reported in vitro to downregulate miR-125b expression and partially restore ATG4D-mediated autophagic flux, thereby improving the stress state and injury phenotype of granulosa cells [200]. Additionally, the upregulation of miR-338-3p has been found to directly target and inhibit PTEN, promoting granulosa cell proliferation and suppressing apoptosis. This effect is thought to potentially contribute to maintaining the stability of the follicular microenvironment that supports oocyte development; however, its direct impact on the oocyte itself remains to be further validated [201]. Evidence from Zhang et al., based on the investigation of the circRHBG/miR-515/SLC7A11 axis in clinical PCOS samples and in vitro granulosa cell models, indicates that serum miR-515-5p has been proposed as a potentially valuable diagnostic biomarker for PCOS, with its expression levels showing a certain correlation with changes in ferroptosis-related molecules, such as SLC7A11 [202]. This finding provides preliminary clues for exploring miRNAs as stratification biomarkers reflecting ferroptosis sensitivity or oxidative stress status. However, the practical application value of these markers in guiding individualized interventions or targeted therapies remains to be systematically evaluated. Overall, current evidence supports the role of miRNAs as a critical molecular layer in regulating homeostatic imbalance and programmed cell death networks in PCOS, highlighting their significant research potential. Nonetheless, existing studies remain primarily at the experimental and correlational stages. Their safety, delivery efficiency, cell-type specificity, and long-term effects must be validated in more rigorous models and clinical trials before they can be translated into practical clinical applications.

#### Epigenetic modifiers

Epigenetic modifiers offer a novel potential pathway for intervening in dysregulated cell death programs in PCOS by modulating post-transcriptional gene expression networks. Recent studies have increasingly focused on the roles of RNA m6A modification and its associated regulatory proteins in ferroptosis and oxidative stress. Mechanistically, the natural naphthoquinone compound Plumbagin (PLB) specifically inhibits the function of the m6A reader protein YTHDF1, thereby blocking its m6A dependent promotion of the translation of SLC7A5, a key ferroptosis-related transporter [196]. In both cellular and animal models, this regulation has been shown to reduce lipid peroxidation levels and attenuate ferroptosis sensitivity, suggesting that it may participate in regulating cell fate within PCOS ovarian tissues by modulating the iron metabolism and amino acid transport axis [196]. On the other hand, inhibitors targeting the RNA demethylase FTO are thought to enhance the stability of GPX4 mRNA—a key antioxidant molecule—thereby improving the cellular capacity to scavenge lipid peroxides. In hyperandrogen-related models, this effect can partially counteract the damage to ovarian tissues caused by oxidative stress and is associated with the attenuation of fibrosis-related signaling pathways [203]. These two epigenetic intervention strategies focus respectively on modulating ferroptosis sensitivity and oxidative stress defense, offering potential therapeutic options for ameliorating pathological ovarian damage in PCOS. However, current evidence is primarily derived from experimental models, and their efficacy and safety in clinical PCOS remain to be further validated.

#### CRISPR/Cas9 gene editing

CRISPR/Cas9 gene editing technology has provided a powerful research tool for dissecting and validating PCOS-related pathogenic signaling pathways. Existing studies have demonstrated that in experimental models, the specific knockout of the transcription factor ETS1 can weaken the abnormal activation of the Androgen Receptor (AR)–NF-κB signaling axis. This, in turn, alleviates the excessively amplified inflammatory cell death responses—including the enhancement of pyroptotic signaling—within the ovarian microenvironment under PCOS pathological conditions [26]. These results support the role of ETS1 as a critical regulator connecting hyperandrogen signaling to the amplification of inflammatory cell death, highlighting its significant value in mechanstic studies of PCOS.In addition to gene-editing approaches, transcriptomic analysis and regulatory network modeling are continuously uncovering new upstream regulatory nodes. Recent research proposes that High Mobility Group AT-Hook 1 (HMGA1) may function as a core transcription factor within the ferroptosis-related regulatory network of granulosa cells, participating in the regulation of oocyte damage associated with oxidative stress and iron metabolism imbalance [204, 205]. These findings provide new molecular clues for understanding the transcriptional regulatory basis of ferroptosis signaling in PCOS. It must be emphasized that current research on ETS1 and HMGA1 is primarily based on cellular and animal models; within this context, CRISPR/Cas9 technology is employed more as a tool for mechanistic validation and functional dissection rather than as a direct clinical intervention strategy. Its safety, specificity, and ethical feasibility in human ovarian tissues remain to be systematically evaluated. Consequently, these research results should be viewed as providing potential targets and a theoretical foundation for future mechanism-oriented intervention explorations, rather than as therapeutic regimens already poised for clinical translation.

To provide a comprehensive overview of the precision therapeutic strategies discussed above, Table 2 summarizes their primary targets, proposed mechanisms of action, supporting evidence, and current stage of investigation in the context of PCOS. The table includes both clinically established interventions and emerging approaches, and serves as a concise reference for understanding how different strategies may modulate inflammation, programmed cell death pathways, epigenetic regulation, and gene regulatory networks, primarily based on experimental, preclinical, and limited clinical evidence.

**Table 2:** Potential modulators targeting cell death and regulatory pathways in PCOS: experimental evidence and developmental status

Therapeutic Agent	Primary Target/Mechanism (Relevance to PCOS)	Type of PCOS Evidence	Key Findings	Development/Clinical Status	Ref.
Metformin	Activates AMPK; reduces mitochondrial ROS; suppresses NLRP3 inflammasome–related signaling	Human + Animal + In vitro	Associated with reduced ovarian inflammatory signaling and improved metabolic parameters; anti–cell death effects in granulosa cells mainly supported by experimental studies	Clinically used for metabolic management in PCOS; ovarian cell death mechanisms largely experimental	[176]
Liraglutide (GLP-1RA)	Modulates NF-κB–related inflammatory signaling and improves metabolic homeostasis	Human (RCT)	Improves body weight, insulin resistance, and hyperandrogenism	Supported by randomized clinical evidence; effects on ovarian cell death pathways are largely indirect	[206]
Semaglutide (GLP-1RA)	Improves metabolic parameters, indirectly reduces oxidative stress and inflammation	Human (clinical cohort)	Significant improvement in weight and metabolic outcomes in PCOS patients	Observational clinical evidence; direct ovarian mechanistic data are lacking	[207]
Curcumin	Suppresses TLR4/NF-κB signaling and attenuates oxidative stress	Human (RCT)	Reduces circulating inflammatory cytokines and androgen levels	Small-scale clinical trials; limited direct evidence on ovarian cell death pathways	[208]
Resveratrol	Inhibits NLRP3 inflammasome activation and NF-κB–IL-1β signaling	Human (RCT)	Improves inflammatory status; effects on the ovarian microenvironment are primarily inferred from experimental models	Limited clinical evidence supplemented by preclinical studies	[209]
Ferrostatin-1	Inhibits lipid peroxidation and GPX4-dependent ferroptosis	Animal (PCOS models)	Alleviates granulosa cell ferroptosis and improves ovarian dysfunction	Preclinical proof-of-concept stage	[210]
MCC950 (NLRP3 inhibitor)	Selectively blocks NLRP3 inflammasome activation	Animal (PCOS models)	Reduces ovarian inflammation and improves follicular development	Preclinical validation stage	[185]
Deferoxamine	Iron chelation; reduces iron-induced oxidative stress and ferroptosis	Animal (PCOS models)	Attenuates ovarian iron overload and restores ovulatory function	Preclinical proof-of-concept stage	[153]
Anti-miR-125b/miR-125b inhibitor	Silences miR-125b and restores ATG4D-mediated autophagic flux	Animal + In vitro	Improves granulosa cell survival and alleviates functional impairment	Preclinical mechanistic studies	[211]
miR-338-3p agonist	Promotes granulosa cell proliferation and suppresses apoptosis via PTEN inhibition	Human serum analysis + In vitro	miR-338-3p is downregulated in PCOS; restoring its expression reduces apoptosis	Exploratory translational research; in vivo therapeutic validation is lacking	[201]
miR-515-5p (biomarker-guided therapy)	Stratifies PCOS patients with SLC7A11-related ferroptosis susceptibility	Human (serum biomarker studies)	Facilitates early identification of ferroptosis-associated PCOS phenotypes	Biomarker discovery stage; therapeutic guidance remains hypothetical	[202]
Plumbagin (PLB)	Inhibits the m6A reader YTHDF1 and reduces ferroptosis susceptibility	Animal (PCOS models)	Decreases ferroptosis-associated ovarian tissue damage	Preclinical mechanistic studies	[196]
FTO inhibitor	Stabilizes GPX4 mRNA and mitigates oxidative stress and ovarian fibrosis	Animal (PCOS models)	Alleviates oxidative stress and fibrosis-related signaling	Preclinical proof-of-concept stage	[212]
CRISPR/Cas9 ETS1 knockout	Reverses AR-NF-κB overactivation, suppresses pyroptosis cascade	Animal/in vitro	Reduces “pyroptosis storm” and ovarian microenvironment damage	Mechanistic research tool; not currently clinically applicable	[26]
HMGA1-targeted regulation	HMGA1 identified as an upstream transcriptional regulator of ferroptosis-related gene networks	Human granulosa cells (omics + bioinformatics)	HMGA1 correlates with ferroptosis-related genes and oocyte qualitycompetence	Target identification stage; direct functional intervention remains to be validated	[213]

The modulators listed above are primarily utilized as experimental tools in preclinical models to validate mechanistic hypotheses. Their clinical application remains exploratory and requires further validation of safety and efficacy in human reproductive health

## Heterogeneity of PCOS and implications for cell death pathways

Polycystic ovary syndrome is increasingly recognized as a heterogeneous condition encompassing diverse metabolic and endocrine phenotypes rather than a single uniform disease entity. Differences between lean and obese individuals, hyperandrogenic and normoandrogenic presentations, and insulin-resistant versus insulin-sensitive subtypes contribute to distinct ovarian microenvironmental states, including variations in inflammatory tone, oxidative stress, and hormonal signaling.

Such heterogeneity is likely to influence the relevance and predominance of regulated cell death pathways. Obesity-associated metabolic stress and chronic low-grade inflammation may favor activation of ferroptotic or pyroptotic signaling, whereas hyperandrogenic states have been more frequently linked to granulosa cell apoptosis and autophagy-related alterations. In contrast, lean phenotypes with limited metabolic disturbance may involve follicular dysregulation driven primarily by endocrine signaling rather than pronounced activation of cell death cascades. These considerations suggest that the engagement of cell fate mechanisms may vary across PCOS subtypes and should not be assumed to be uniform.

Nevertheless, direct comparative evidence connecting specific PCOS phenotypes to differential activation of cell death modalities remains limited. Many experimental studies do not stratify samples according to metabolic or hormonal subtype, restricting mechanistic resolution. Future investigations incorporating phenotype-specific designs will be essential to refine translational relevance and to determine whether cell death signatures could contribute to clinically meaningful stratification.

## Current limitations and ongoing debates

Despite the integrative perspective proposed in this review, several limitations should be acknowledged. Polycystic ovary syndrome is a heterogeneous and multifactorial disorder, and follicular developmental arrest cannot be fully explained by regulated cell death mechanisms alone. Endocrine dysregulation, including altered gonadotropin signaling, hyperandrogenism, insulin resistance, and metabolic imbalance, contributes substantially to ovarian dysfunction through pathways that may not primarily involve cell death processes.

Furthermore, experimental evidence linking specific cell death modalities to PCOS remains uneven. While apoptosis has been relatively well studied, mechanistic data for pyroptosis, necroptosis, and ferroptosis are limited, and findings across model systems are not always consistent. In several cases, pathway interactions are inferred from non-ovarian or non-PCOS contexts, highlighting the need for cautious interpretation.

Therefore, the proposed death crosstalk network should be viewed as a conceptual framework intended to stimulate hypothesis-driven investigation rather than a definitive mechanistic model. Future work integrating endocrine, metabolic, and cell fate regulatory perspectives will be essential for a more comprehensive understanding of follicular pathophysiology in PCOS.

## Conclusion

Collectively, this review constructs a conceptual framework—the ‘Death Crosstalk Network’ model—which integrates multiple programmed cell death pathways into a dynamic regulatory system to explain the potential mechanisms underlying follicular dysfunction in PCOS. This integrated model provides a novel systemic perspective on how hyperandrogenism, oxidative stress, and metabolic dysregulation converge at the level of cell fate regulation. Beyond synthesizing existing evidence, this review proposes a system-level hypothesis aimed at providing a theoretical foundation for future research exploring the synergistic regulation of ovarian microenvironment homeostasis across multiple pathways.

The pathophysiological process of PCOS may be largely influenced by the imbalance of the ovarian microenvironment, in which the abnormal activation of multiple programmed cell death pathways is thought to form a dynamically interacting network. Among the upstream drivers, hyperandrogenism may activate inflammatory responses and pyroptotic processes via the nuclear factor-κB (NF-κB)–NLRP3 inflammasome axis. Meanwhile, insulin resistance-related oxidative stress leads to the accumulation of reactive oxygen species (ROS), synergistically triggering apoptosis, ferroptosis, and autophagic dysfunction. Metabolic disturbances, such as lipotoxicity, further amplify these death signals [214]. The central role of the death crosstalk hub is manifested as follows: inhibition of caspase-8 activity blocks apoptosis, releasing RIPK1- and RIPK3 kinase-dependent necroptosis and pyroptosis signals [215]; Gasdermin family members GSDMD and GSDME integrate pyroptotic execution, apoptosis-to-pyroptosis conversion, and ferroptosis signaling, mediating inflammatory propagation and ovarian fibrosis [30]; endoplasmic reticulum stress and iron metabolic imbalance synergistically promote apoptosis and ferroptosis through the CHOP-PUMA signaling axis and p53-dependent suppression of SLC7A11 [216]; mitochondrial dysfunction serves as a comorbid pathological platform for apoptosis mediated by mitochondrial outer membrane permeabilization and lipid peroxidation-driven ferroptosis [217].

This death crosstalk may establish a self-reinforcing pathological cascade, whereby granulosa cell loss could accelerate follicular atresia, ovarian fibrosis, and insulin resistance, potentially feeding back to intensify hyperandrogenism and metabolic disorders. Based on this model, current research proposes various regulatory pathways that may influence the amplification of death signals. These include modulating AMPK-related pathways through metabolic regulation and anti-inflammatory effects, indirectly alleviating cellular damage by improving insulin resistance and inflammatory status, or influencing ovarian microenvironment homeostasis by targeting the interaction nodes between ferroptosis and inflammatory signaling. Meanwhile, studies focusing on molecular nodes such as the NLRP3 inflammasome, RIPK1/RIPK3 kinases, and the Hippo pathway effector YAP serve primarily as tools for mechanistic exploration to dissect the potential patterns of death pathway cross-regulation.At a more refined regulatory level, miRNAs, RNA epigenetic modifications, and associated metabolic pathways have been proposed to participate in modulating cell death sensitivity; however, most of these studies remain at the experimental and correlational stages. It must be emphasized that the various regulatory mechanisms discussed in this review are primarily inferred from cellular and animal models and have not yet received direct clinical validation in human PCOS tissues. This review does not advocate for the direct application of cell death pathway modulation to clinical treatment at the current stage. Instead, by proposing the conceptual ‘Death Crosstalk Network’ model, it aims to provide a theoretical foundation for subsequent mechanistic validation in human PCOS samples and the cautious exploration of potential translational directions.

## Future perspectives

This review has discussed how the death crosstalk network, through dynamic interactions among apoptosis, pyroptosis, necroptosis, autophagy, and ferroptosis, constitutes the central pathological hub underlying follicular developmental disorders in polycystic ovary syndrome (PCOS). It should be noted that the mechanistic insights summarized in this review are largely drawn from animal or in vitro models, and direct evidence in human PCOS remains limited. Therefore, the relevant conclusions should be viewed as a hypothesis-generating synthesis rather than a clinically proven mechanistic framework.The primary limitation of current research lies in the fact that the spatiotemporal specificity of cell death signaling cross-regulation has not yet been systematically resolved. Across different follicular developmental stages, granulosa cells and theca cells may exhibit differentiated biases in cell death patterns. However, most existing studies are still based on single time points or isolated cell types, making it difficult to reflect the true dynamic evolution of death signals within the PCOS ovarian microenvironment.Secondly, research regarding the interactions between different cell types remains insufficient.

The transmission of death signals between local ovarian immune cells, stromal cells, and granulosa cells—mediated by Damage-Associated Molecular Patterns (DAMPs) and inflammatory mediators—may play a key role in amplifying follicular damage. Nevertheless, direct evidence supporting the specific molecular mechanisms of this process in PCOS is still lacking. Furthermore, the systemic effects of the ‘Death Crosstalk Network’ need to be understood from a cross-organ perspective. Ovarian-derived inflammation and cell damage signals may influence the liver, adipose tissue, and insulin sensitivity through circulating factors, thereby back-feeding and exacerbating hyperandrogenism and metabolic disorders; however, these causal relationships have not yet been fully validated. From the perspective of translational research, exploring the targeted regulation of death signaling crosstalk still faces significant challenges. On one hand, multiple programmed cell death pathways play dual roles in maintaining physiological homeostasis, and non-selective interventions may lead to unpredictable side effects. On the other hand, strategies for ovary-specific delivery and concerns regarding long-term safety remain to be addressed. Consequently, this review does not advocate for the direct clinical application of cell death pathway modulation at the current stage.

Future research should focus on: validating the existence and molecular characteristics of the Death Crosstalk Network in human PCOS ovarian tissues; elucidating the heterogeneous basis of death signal imbalance across different PCOS subtypes; and reconstructing the dynamic regulatory landscape of cell death signaling within the follicular microenvironment through multi-omics and spatial biology technologies. As these basic and translational studies progress, the Death Crosstalk Network is expected to become a vital conceptual framework for deepening our understanding of PCOS pathogenesis and providing a theoretical foundation for exploring subsequent mechanism-oriented intervention strategies.

## Data Availability

No datasets were generated or analysed during the current study.
